# LiGe(SiMe_3_)_3_: A New Substituent for the Synthesis of Metalloid Tin Clusters from Metastable Sn(I) Halide Solutions

**DOI:** 10.3390/molecules23051022

**Published:** 2018-04-26

**Authors:** Mareike Binder, Claudio Schrenk, Theresa Block, Rainer Pöttgen, Andreas Schnepf

**Affiliations:** 1 Institut für Anorganische Chemie, Universität Tübingen, Auf der Morgenstelle 18, D-72076 Tübingen, Germany; mareike.binder@uni-tuebingen.de (M.B.); claudio.schrenk@uni-tuebingen.de (C.S.); 2 Institut für Anorganische und Analytische Chemie, Universität Münster, Corrensstrasse 30, D-48149 Münster, Germany; t_bloc02@uni-muenster.de (T.B.); pottgen@uni-muenster.de (R.P.)

**Keywords:** co-condensation, disproportionation, germanium, tin, Mössbauer spectroscopy, bulky substituents, metalloid clusters, nanoscaled clusters

## Abstract

The most fruitful synthetic route to metalloid tin clusters applies the disproportionation reaction of metastable Sn(I) halide solutions, whereby Si(SiMe_3_)_3_ is mostly used as the stabilizing substituent. Here, we describe the synthesis and application of the slightly modified substituent Ge(SiMe_3_)_3_, which can be used for the synthesis of metalloid tin clusters to give the neutral cluster Sn_10_[Ge(SiMe_3_)_3_]_6_ as well as the charged clusters {Sn_10_[Ge(SiMe_3_)_3_]_5_}^−^ and {Sn_10_[Ge(SiMe_3_)_3_]_4_}^2−^. The obtained metalloid clusters are structurally similar to their Si(SiMe_3_)_3_ derivatives. However, differences with respect to the stability in solution are observed. Additionally, a different electronic situation for the tin atoms is realized as shown by ^119m^Sn Mössbauer spectroscopy, giving further insight into the different kinds of tin atoms within the metalloid cluster {Sn_10_[Ge(SiMe_3_)_3_]_4_}^2−^. The synthesis of diverse derivatives gives the opportunity to check the influence of the substituent for further investigations of metalloid tin cluster compounds.

## 1. Introduction

In the last couple of years, the developments in nanotechnology as well as their industrial applications have generated interest in nanoscaled molecular materials [1]. One example of such nanoscaled compounds is the group of metalloid clusters of the general formula M_n_R_m_ (n > m; M = Al, Ga, Sn, Pb, Au, etc.; R = N(SiMe_3_)_2_, Si(SiMe_3_)_3_, etc.) [2]. As the average oxidation state of the metal atoms within such metalloid clusters is between zero and one, these clusters are localized on the border between molecules and the solid state of metals. Consequently, beside a technological interest, also fundamental questions might be addressed by such metalloid cluster compounds [3,4,5].

In the case of metalloid cluster compounds of tin, we introduced a special synthetic route, applying the disproportionation reaction of a metastable Sn^I^ halide solution [6,7]. The metastable solution is obtained via a preparative co-condensation technique. Thereby, a Sn^I^ halide is condensed together with a mixture of an organic solvent and a donor component such as P*n*Bu_3_ at −196 °C. This leads to a solid matrix, where the monohalides are completely separated from each other by the solvent molecules. After heating the solid matrix to −78 °C, a metastable solution of Sn^I^ halides is obtained, if the right solvent–donor mixture is used during the co-condensation. Starting from these metastable solutions, we were able to establish a synthetic route to metalloid tin clusters in recent years, whereby it is important to find a suitable substituent which stabilizes the metastable clusters.

The last years have shown that in particular, the bulky Si(SiMe_3_)_3_ [Hyp] substituent is very useful to trap tin-rich metalloid clusters before complete disproportionation to elemental tin occurs. In this way, a variety of clusters with nine or ten tin atoms in the cluster core could be obtained [8,9,10,11,12,13,14], for example, [Sn_9_(Hyp)_2_]^2−^ [15], [Sn_9_(Hyp)_3_]^−^ [16], [Sn_10_(Hyp)_4_]^2−^ [17], [Sn_10_(Hyp)_5_]^−^ [18], and Sn_10_(Hyp)_6_ [19]. Especially [Sn_9_(Hyp)_2_]^2−^, [Sn_9_(Hyp)_3_]^−^, and [Sn_10_(Hyp)_4_]^2−^ are of particular interest, since these clusters exhibit an open ligand shell in which the tin atoms are incompletely shielded by the bulky Hyp ligands and can be attacked by Lewis acids. For example, recent investigations with the metalloid tin cluster [Sn_10_(Hyp)_4_]^2−^ have shown that the reaction with (Ph_3_P)Au-S(Hyp) [20] undergoes a complicated scrambling of the metal atoms, which results in the intermetalloid cluster [Au_3_Sn_18_(Hyp)_8_]^−^. This cluster is the longest intermetalloid chain compound of tin found to date [21]. Furthermore, [Sn_10_(Hyp)_3_]^−^ is formed during the reaction of [Sn_10_(Hyp)_4_]^2−^ with ZnCl_2_ by the elimination of ZnCl(Hyp) [22].

While the syntheses with the Hyp substituent work relatively well, and the resulting clusters are also useful for further investigations, we wondered if a change to a similar substituent might lead to new metalloid clusters with a similar or different size and structure. Herein, we present the first results applying the germanide [Ge(SiMe_3_)_3_]^−^ as a substituent during the synthesis of metalloid tin clusters via the disproportionation reaction of Sn^I^ halides.

## 2. Results and Discussion

The germanide [Ge(SiMe_3_)_3_]^−^ is synthesized by a similar synthetic route to that of the (Hyp) substituent [23]. Hence, first of all, Me_3_SiCl and GeCl_4_ are coupled in the presence of lithium to give Ge(SiMe_3_)_4_ in 72% yield. Afterwards, the germane Ge(SiMe_3_)_4_ is reacted with methyllithium in THF at room temperature for several days, to give the anticipated compound LiGe(SiMe_3_)_3_ ∙ 2.75 THF in 81% yield after recrystallization.

### 2.1. Sn_10_(Ge(SiMe_3_)_3_)_6_ ***1***

**Figure 1:**
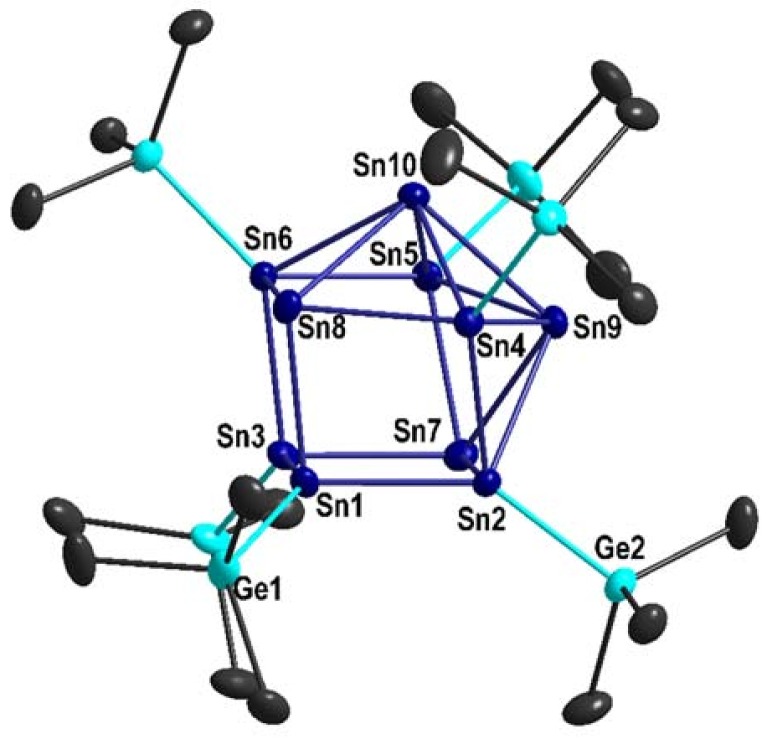
Molecular structure of Sn_10_[Ge(SiMe_3_)_3_]_6_ **1**, without CH_3_ groups for clarity (displacement ellipsoids with 50% probability). Selected bond lengths [pm] and angles [°]: Sn1-Sn2: 292.16(3); Sn1-Sn3: 284.85(3); Sn1-Sn8: 288.51(3); Sn2-Sn4: 295.48(3); Sn2-Sn7: 301.94(3); Sn2-Sn9: 304.13(3); Sn3-Sn6: 291.25(3); Sn3-Sn7: 287.32(4); Sn4-Sn8: 292.88(3); Sn4-Sn9: 300.76(3); Sn4-Sn10: 298.77(3); Sn5-Sn6: 294.52(3); Sn5-Sn7: 293.02(3); Sn5-Sn9: 298.53(3); Sn5-Sn10: 301.42(3); Sn6-Sn8: 302.72(3); Sn6-Sn10: 303.42(3); Sn7-Sn9: 310.80(3); Sn8-Sn10: 311.68(3); Sn9-Sn10: 307.29(3); Sn1-Ge1: 266.17(4); Sn2-Ge2: 268.99(4); Sn1-Sn2-Sn7: 89.455(9); Sn2-Sn1-Sn3: 89.500(9); Sn2-Sn1-Sn8: 97.195(10); Sn1-Sn2-Sn4: 80.733(9); Sn2-Sn4-Sn10: 106.176(9); Sn8-Sn4-Sn9: 108.278(10); Ge1-Sn1-Sn8: 108.249(14); Ge2-Sn2-Sn7: 105.781(12).

After the successful synthesis of LiGe(SiMe_3_)_3_, it was necessary to check if LiGe(SiMe_3_)_3_ can be used for the synthesis of metalloid tin clusters via the disproportionation reaction of a metastable Sn(I) halide solution. For this purpose, we reacted a −78 °C cold Sn^I^Cl solution (0.2 M) with LiGe(SiMe_3_)_3_ ∙ 2.75 THF. The reaction mixture is slowly warmed to room temperature to give a black-colored reaction solution without any elemental tin precipitates, indicating that the anticipated trapping of intermediates on the way to elemental tin was successful. After some work-up procedures, black crystals of Sn_10_[Ge(SiMe_3_)_3_]_6_ **1** could be obtained at −30 °C from a pentane solution. The molecular structure of **1** is presented in Figure 1, showing that **1** is a metalloid Sn cluster in which the ten tin atoms are arranged in the form of a centaur polyhedron, whereby six Ge(SiMe_3_)_3_ substituents are bound to the cluster core. **1** is thus isostructural to Sn_10_(Hyp)_6_, indicating that the substitution of Si(SiMe_3_)_3_ by Ge(SiMe_3_)_3_ does not lead to a different arrangement of the tin atoms in the cluster and that in both cases, the cluster core is completely shielded by the six substituents.

This result directly shows that the Ge(SiMe_3_)_3_ substituent can be used for the synthesis of metalloid tin clusters. Furthermore, **1** is soluble in common organic solvents, such as toluene, THF, and so forth, which is due to its almost completely shielded tin core giving a nearly perfect nonpolar sphere.

As shown in Table 1, the bond distances within **1** fit very well to the bond distances of the already described Sn_10_(Hyp)_6_. This shows that also within **1**, two different types of tin atoms are present inside the metalloid cluster, where the average tin–tin distance in the cubic part of the centaur polyhedron (291 pm) fits to the tin–tin distance found in α-tin (281 pm), while the average tin–tin distance in the icosahedral part of the centaur polyhedron (305 pm) is comparable to the average tin–tin distance found in β-tin (307 pm).

The similar tin–tin distances in **1** and Sn_10_(Hyp)_6_ show that the larger Ge(SiMe_3_)_3_ substituent has a similar bulkiness to the smaller Hyp substituent. This might be due to the fact that although Ge(SiMe_3_)_3_ is larger than Hyp, it is also bound farther away from the cluster core due to the longer average Ge–Sn distance of 267.3 pm in **1** with respect to the average Si–Sn distance of 264.2 pm in Sn_10_(Hyp)_6_, and both effects compensate each other. However, besides this steric similarity, an electronic difference might be present due to the different electronegativities of Si and Ge.

**Figure 2:**
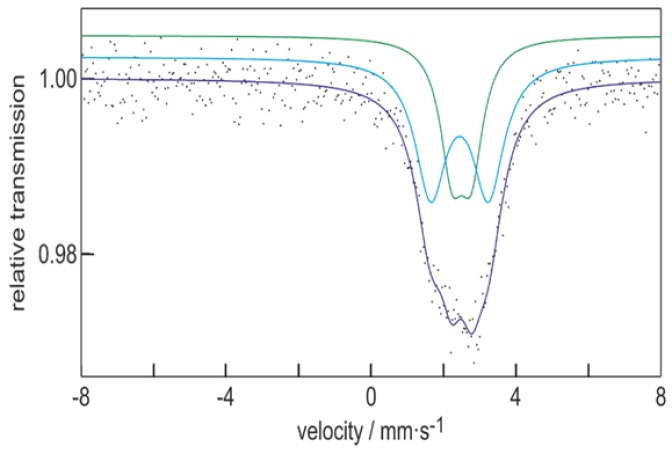
Experimental (data points) and simulated (continuous lines) ^119m^Sn Mössbauer spectrum of Sn_10_[Ge(SiMe_3_)_3_]_6_ **1** at 5 K. For the fitting parameters, see Table 2.

To get a first insight into the electronic situation of **1**, we performed quantum chemical calculations [24,25,26,27,28,29,30,31,32,33], whereby similar shared electron numbers (SENs) [34] are calculated for **1** as already obtained for Sn_10_(Hyp)_6_, showing that a similar bonding is present inside the cluster. However, the calculated charges of the tin atoms within **1** are very similar, only ranging from +0.12 to −0.11. In the case of Sn_10_(Hyp)_6_, the calculated charges show a larger deviation, ranging from −0.51 to +0.25. Hence, by changing the ligand from Si(SiMe_3_)_3_ to Ge(SiMe_3_)_3_, the tin atoms inside Sn_10_R_6_ become electronically more similar. This finding is supported experimentally by results of Mössbauer spectroscopy; that is, the ^119m^Sn Mössbauer spectrum of **1** (Figure 2) shows two quadrupole-split signals A1 and A2 at isomer shifts of *δ* = 2.45(2) (with Δ*E*_Q_ = 1.59(5) mm s^−1^ and *Γ* = 1.05(4) mm s^−1^) and *δ* = 2.51(2) (with Δ*E*_Q_ = 0.54(5) mm s^−1^ and *Γ* = 0.79(4) mm s^−1^) in a fixed area ratio of 60:40 (fitting parameters are given in Table 2). It needs to be said that the area was fixed to avoid correlation of the quadrupole splitting and the experimental line width; even without fixing, approximately identical results could be received. These two signals of **1** might be assigned to the ligand bound and the naked tin atoms where a ratio of 6:4 is present. Another possibility whereby to describe a 6:4 ratio is realized by dividing the tin atoms in the cluster in those localized in the cubic part and those localized in the icosahedral part of the centaur polyhedron.

Similar assignments were already done for Sn_10_(Hyp)_6_ [19], where the isomer shifts are 2.35(1) and 2.56(1) mm s^−1^. Consequently, in the case of **1**, the two signals within the spectrum differ by 0.06 mm s^−1^, while in the case of Sn_10_(Hyp)_6_, the difference of 0.21 mm s^−1^ is much larger, being in line with the calculated differences of the charges of the tin atoms inside the cluster. This result shows that the different ligand indeed changes the electronic situation inside the cluster, although the structure remains similar. Additionally, the chemical behavior can be very similar, as seen in the following.

### 2.2. Li(thf)_4_Sn_10_[Ge(SiMe_3_)_3_]_5_ ***2***

**Figure 3:**
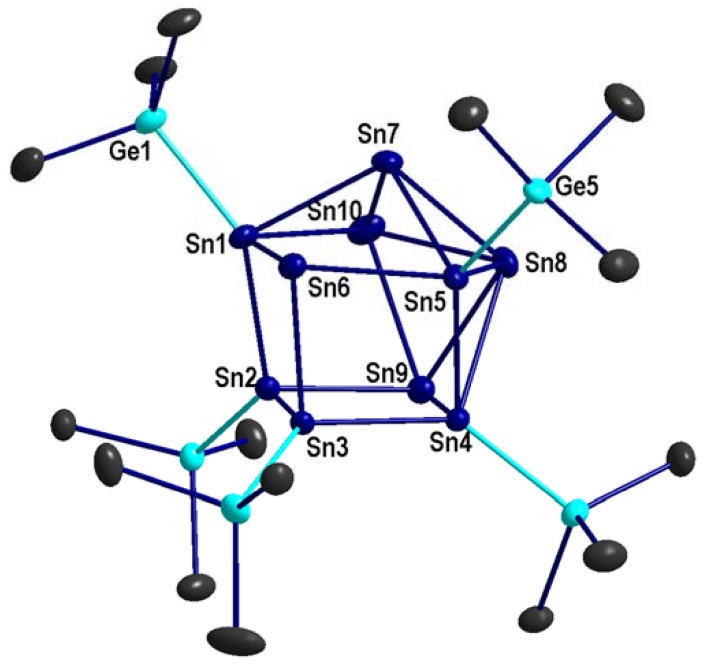
Molecular structure of the anionic metalloid cluster {Sn_10_[Ge(SiMe_3_)_3_]_5_}^−^, without CH_3_ groups (displacement ellipsoids with 50% probability). Selected bond lengths [pm] and angles [°]: Sn1-Sn2: 289.46(2); Sn1-Sn6: 294.95(2); Sn1-Sn7: 295.34(3); Sn1-Sn10: 301.15(3); Sn2-Sn3: 293.69(2); Sn2-Sn9: 287.66(2); Sn3-Sn4: 291.84(2); Sn3-Sn6: 294.31(2); Sn4-Sn5: 292.10(2); Sn4-Sn8: 325.57(3); Sn4-Sn9: 289.73(2); Sn5-Sn6: 294.15(2); Sn5-Sn7: 294.59(3); Sn5-Sn8: 302.91(3); Sn6-Sn7: 329.52(3); Sn7-Sn8: 299.29(3); Sn7-Sn10: 298.05(3); Sn8-Sn9: 303.20(3); Sn8-Sn10: 296.69(3); Sn9-Sn10: 301.88(3); Sn1-Ge1 266.90(3); Sn5-Ge5: 266.62(3); Sn1-Sn2-Sn3: 84.798(6); Sn2-Sn3-Sn4: 83.593(6); Sn2-Sn3-Sn6: 95.120(6); Sn3-Sn4-Sn5: 81.171(6); Sn3-Sn4-Sn8: 113.880(7); Sn4-Sn5-Sn7: 108.572(7); Sn6-Sn5-Sn8: 114.487(8); Sn7-Sn5-Sn8: 60.100(7); Sn7-Sn10-Sn9: 106.442(7); Sn7-Sn10-Sn8: 60.427(7); Ge1-Sn1-Sn6: 115.335(9); Ge5-Sn5-Sn6: 114.513(9).

Another metalloid tin cluster that could be achieved by the reaction of the metastable Sn^I^Cl solution with LiGe(SiMe_3_)_3_ ∙ 2.75 THF is Li(thf)_4_Sn_10_[Ge(SiMe_3_)_3_]_5_ **2**, which is obtained as black crystals from a toluene extract after diverse work-up procedures. The molecular structure of the metalloid cluster anion {Sn_10_[Ge(SiMe_3_)_3_]_5_}^−^ in **2** is shown in Figure 3, again exhibiting a similar arrangement of the tin atoms in the cluster core as found in the corresponding Hyp derivative [Sn_10_(Hyp)_5_]^−^ (Table 3). As well as the comparable structure, the chemical behavior is also similar, as **2** decomposes upon dissolving, hindering further characterization via NMR or electrospray mass spectrometry. However, in the solid state, the compound seems to be stable as the composition of the crystals does not change by time, as shown by energy-dispersive X-ray (EDX) spectroscopy (Supplementary Materials, Figure S11, Table S1).

As the behavior of the so-far synthesized metalloid tin clusters, as well as the synthesis, is comparable if Hyp or Ge(SiMe_3_)_3_ is used, we wondered if also the high-yield synthesis of the anionic cluster [Sn_10_(Hyp)_4_]^2-^ with its open ligand shell might be possible, applying Ge(SiMe_3_)_3_ as the substituent as discussed in the following.

### 2.3. [Li(tmeda)_2_]_2_Sn_10_[Ge(SiMe_3_)_3_]_4_ ***3***

**Figure 4:**
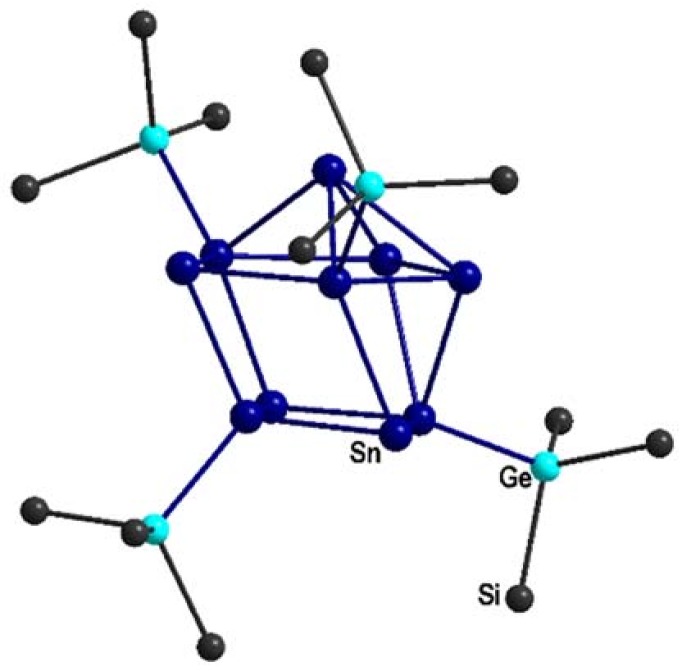
Connectivity map of the cluster core of the anion {Sn_10_[Ge(SiMe_3_)_3_]_4_}^2−^.

To obtain the anticipated product, we changed the synthetic procedure accordingly. This time, the reaction solution is filtered to remove the metathesis salt, and afterwards, tetramethylethylenediamine (TMEDA) is added as a complexing reagent. In doing so, after a couple of days, black crystals of [Li(TMEDA)_2_]_2_Sn_10_[Ge(SiMe_3_)_3_]_4_ **3** could be obtained, showing similar crystallographic problems to the Hyp derivative that hinder a proper structure solution. Hence, the black cubic crystals of **3**, obtained by adding TMEDA as a complexing agent, only diffract well to a 2θ angle of 20°. Recrystallization in the presence of the complexing reagent 12-crown-4 increases the crystal size, but not the crystal quality, and thus the crystal structure could not be solved properly, and only the positions of the Sn, Ge, and Si atoms are refined (Figure 4) [35]. However, the rudimentarily solved crystal structure already shows that the arrangement within the anion {Sn_10_[Ge(SiMe_3_)_3_]_4_}^2−^ in **3** is similar to [Sn_10_Hyp_4_]^2−^ (a comparison of the different tin–tin distances can be found in the Supplementary Materials, Table S2).

Even though **3** is structurally similar to the Hyp derivative [Sn_10_(Hyp)_4_]^2−^, the electronic and chemical characteristics are different. This means that although {Sn_10_[Ge(SiMe_3_)_3_]_4_}^2−^ decomposes like [Sn_10_(Hyp)_4_]^2−^ after dissolving the crystals in THF, this time the decomposition is slower and only after one day at room temperature, a metallic luster on the NMR tube’s surface and a black precipitate is observed. The ^1^H-NMR of freshly dissolved crystals shows a singlet at 0.36 ppm and already some signals of the decomposition products at 0.30 ppm and 0.25 ppm. However, as the decomposition of **3** is slower than the one of the corresponding [Sn_10_(Hyp)_4_]^2−^, it was possible to obtain ^13^C- and ^29^Si-NMR data of **3** as well.

**Figure 5:**
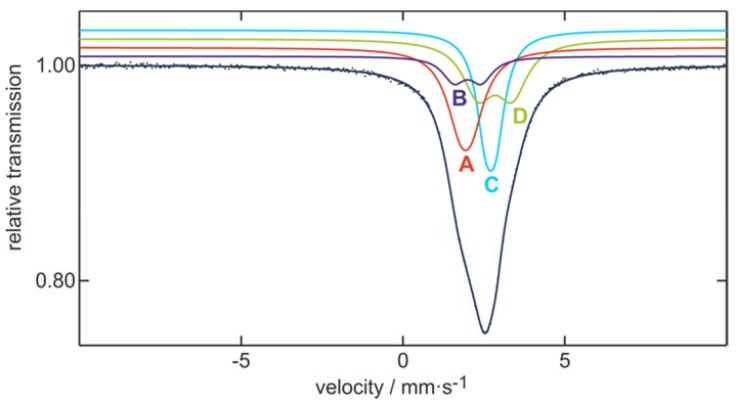
Experimental (data points) and simulated (continuous lines) ^119m^Sn Mössbauer spectrum of {Sn_10_[Ge(SiMe_3_)_3_]_4_}^2–^ at 6 K. For the fitting parameters, see Table 4.

**Figure 6:**
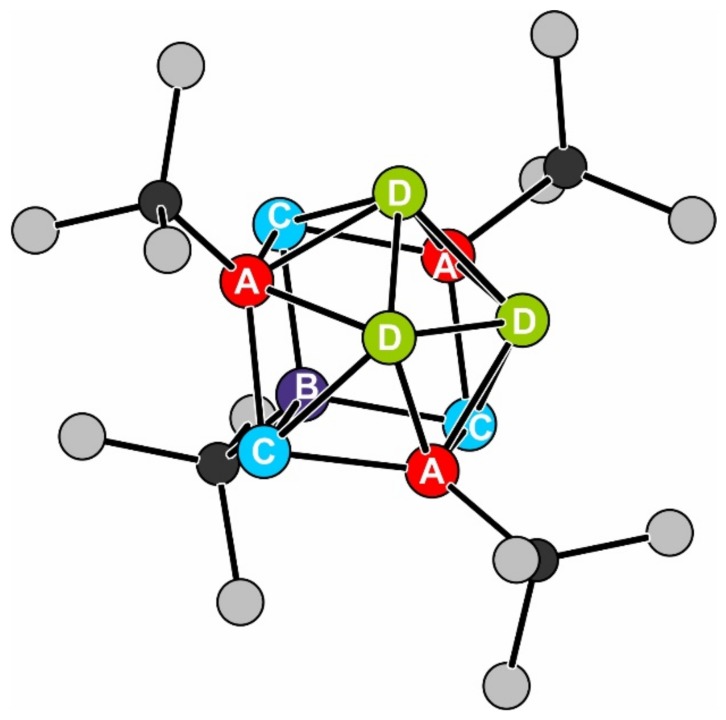
The cluster core of {Sn_10_[Ge(SiMe_3_)_3_]_4_}^2−^. The different colors of the tin sites correspond to the groups listed in Table 4. For details, see text.

To obtain further information about the differences in both [Sn_10_R_4_]^2−^ clusters, an ^119m^Sn Mössbauer spectrum of **3** is collected. The ^119m^Sn Mössbauer spectrum of **3** is presented in Figure 5 along with the transmission integral fit. The corresponding fitting parameters for **3** are summarized in Table 4. The cluster shows ten Sn atoms on four different sites (Sn(A), Sn(B), Sn(C), and Sn(D); Figure 6) with different calculated partial charges as obtained from quantum chemical calculations (*vide ultra*). These charges along with the crystallographic information were used to regroup the ten tin atoms in **3**.

Compared to [Sn_10_(Hyp)_4_]^2−^, showing only one signal for the ten tin atoms at an isomer shift of δ = 2.50(1) mm s^−1^, the spectrum of **3** could be well reproduced with four different signals with a fixed area ratio of 1:3:3:3. All four subsignals show isomer shifts between 1.93(2) and 2.85(4) mm s^–1^, which is in the typical range for Sn(0) species [36] and in very good agreement with recently reported ^119m^Sn Mössbauer spectroscopic data on similar Sn-cluster compounds such as [Sn_9_(Hyp)_2_]^2–^ [15], Sn_10_[Hyp]_6_ [19], [Sn_10_(Hyp)_4_]^2–^ [17], and [Sn_10_(Hyp)_3_]^–^ [22]. Figure 6 shows the Sn_10_(GeSi_3_)_4_ core of {Sn_10_[Ge(SiMe_3_)_3_]_4_}^2−^, whereby the four different colors represent the groups of tin atoms which are responsible for the different subsignals.

Signal A shows an isomer shift of 1.93(2) mm s^–1^ and a small quadrupole splitting of 0.36(7) mm s^–1^. This signal is caused by the red-colored (Figure 6) substituent bound tin atoms Sn(A). The Ge–Sn bond leads to a decreased electron density in comparison to the other Sn atoms. The Sn(A) atoms have four Sn neighbors.

The fourth substituent bound tin atom Sn(B) (dark blue in Figure 6) is just binding to three Sn atoms. This leads to a small difference in the electron density, and results in a slightly increased isomer shift of 2.00(7) mm s^–1^ (Signal B). The higher quadrupole splitting of 0.8(1) mm s^–1^ is caused by the more asymmetric coordination.

The third signal (C) can be assigned to the light-blue-colored tin atoms of the site Sn(C) with an isomer shift of 2.712(6) mm s^–1^. The small quadrupole splitting of 0.31(1) mm s^–1^ can be explained by the relatively symmetric coordination by the other Sn atoms in the cluster. The Sn(C) atoms are not bound to a substituent, which leads to a higher electron density in comparison with the tin atoms of the sites Sn(A) and Sn(B).

The green-colored Sn atoms of the site Sn(D) form a tin triangle which has no substituent contacts. The triangle is moved slightly out of the cluster center, which is evident from the Sn bond lengths. Between the Sn atoms from the sites Sn(A) to Sn(C), the Sn–Sn distances are around 290 to 294 pm; bonds to and between the tin atoms of site Sn(D) are longer and about 303 pm. This causes an even higher electron density and thus a higher isomer shift of 2.85(4) mm s^–1^ and a quadrupole splitting of 1.04(6) mm s^–1^ (Signal D). The slightly enhanced line width of signals A and D is caused by the superposition of different tin sites. The small differences cannot be resolved.

## 3. Materials and Methods

All reactions were carried out under rigorous exclusion of air and moisture using standard Schlenk techniques under nitrogen atmosphere. All solvents were dried and purified by standard procedures.

### 3.1. LiGe(SiMe_3_)_3_

LiGe(SiMe_3_)_3_ is synthesized by a similar synthetic route to that of the (Hyp) substituent [23]. Therefore, 15.5 mL (24.8 mmol, 1.1 eq) methyllithium solution (1.6 M in Et_2_O) diluted in 50 mL THF was added dropwise to 8.33 g (22.8 mmol, 1 eq) Ge(SiMe_3_)_4_ dissolved in 100 mL THF. The reaction mixture was stirred for 72 h at room temperature and the solvent was removed in vacuo. The remaining white residue was recrystallized in Et_2_O at −30 °C, whereby pale-yellow crystals of LiGe(SiMe_3_)_3_ · 2.75 THF were obtained; yield: 4.68 g (9.4 mmol, 41%). The amount of 2.75 THF is determined via the peak integral ratio in proton NMR compared to that of the SiMe_3_ groups. ^1^H-NMR (250 MHz, C_6_D_6_): δ = 0.65 (s, 27 H, Si*Me_3_*), 1.35 (m, 11 H, *CH_2_*), 3.48 ppm (m, 11 H, O*CH_2_*). ^13^C{^1^H}-NMR (63 MHZ, C_6_D_6_): δ = 7.79 (s, Si*Me_3_*), 25.96 (s, *CH_2_*), 68.65 ppm (s, O*CH_2_*). ^29^Si-NMR (50 MHZ, C_6_D_6_): δ = −3.03–−4.71 ppm (m, ^2^*J*_Si-H_ = 6.0 Hz, *Si*Me_3_).

### 3.2. Sn_10_(Ge(SiMe_3_)_3_)_6_ (***1***)

20 mL of a −78 °C cold 0.2 M solution of Sn^I^Cl in toluene/P*n*Bu_3_ (volume ratio 10:1; 4 mmol) was added to the −78 °C precooled solid LiGe(SiMe_3_)_3_ ∙ 2.75 THF (2.19 g; 4.4 mmol). The reaction mixture was allowed to reach room temperature overnight. The resulting black solution was filtered from the white precipitate and was dried in vacuo. The remaining oil was extracted in pentane, whereby at −30 °C black crystalline blocks of Sn_10_[Ge(SiMe_3_)_3_]_6_ were obtained from the pentane extract; yield: 109 mg (10%). ^1^H-NMR (250 MHz, C_6_D_6_): δ = 0.62 ppm (s, 162H); ^13^C{^1^H}-NMR (63 MHz, C_6_D_6_) δ = 6.54 ppm (s; SiMe_3_); ^29^Si-NMR (50 MHz, C_6_D_6_) δ = −0.13–−1.29 ppm (decet, ^2^*J*_Si-H_ = 6.5 Hz, *Si*Me_3_); elemental analysis calc (%) for Sn_10_Ge_6_Si_18_C_64_H_186_ M_w_ = 3085.4 g mol^−1^: C 24.9, H 6.0; found: C 24.7, H 6.0.

### 3.3. [Li(thf)_4_]{Sn_10_[Ge(SiMe_3_)_3_]_5_} (***2***)

20 mL of a −78 °C cold 0.2 M solution of Sn^I^Cl in toluene/P*n*Bu_3_ (volume ratio 10:1; 4 mmol) was added to the −78 °C precooled solid LiGe(SiMe_3_)_3_ ∙ 2.75 THF (2.19 g; 4.4 mmol). The reaction mixture was allowed to reach room temperature overnight to give a black reaction solution. The reaction solution is then filtered from the white precipitate and dried in vacuo to give a black oily residue. The residue was extracted by pentane to give a black pentane extract. On cooling the pentane extract to −30 °C, a black oil precipitated. The solution was filtered and the oil was dissolved in toluene. Storing the black toluene solution at 6 °C gives black hexagonal crystals of Li(thf)_4_Sn_10_(Ge(SiMe_3_)_3_)_5_; yield: 48 mg (4%). EDX: calc. (mass %) for Ge_5_Si_15_Sn_10_; M_w_ = 1971.59 g mol^−1^: Sn 60.2, Ge 18.4, Si 21.4; found: Sn 56.9; Ge 20.8, Si 22.3. NMR: not possible, due to decomposition of the product in solution.

### 3.4. [Li(TMEDA)_2_]_2_{Sn_10_[Ge(SiMe_3_)_3_]_4_} (***3***)

20 mL of a −78 °C cold 0.2 M solution of Sn^I^Cl in toluene/P*n*Bu_3_ (volume ratio 10:1; 4 mmol) was added to the −78 °C precooled solid LiGe(SiMe_3_)_3_ ∙ 2.75 THF (2.19 g; 4.4 mmol). The reaction mixture was allowed to reach room temperature overnight to give a black reaction solution. The solution was filtered from the white precipitate and TMEDA (0.2 mL) was added. After 12 h, the solution was filtered from a black crystalline precipitate, which was identified as [Li(tmeda)_2_]_2_Sn_10_[Ge(SiMe_3_)_3_]_4_ by single-crystal X-ray diffraction and NMR spectroscopy; yield: 249 mg (22%). These crystals (200 mg, 0.075 mmol) were dissolved in THF at −78 °C and treated with a solution (0.2 mL) of 12-crown-4 in toluene. After a few days at −30 °C, crystals of the metalloid cluster compound [Li(12-crown-4)_2_]_2_Sn_10_[Ge(SiMe_3_)_3_]_4_ were obtained.

NMR data of [Li(tmeda)_2_]_2_Sn_10_[Ge(SiMe_3_)_3_]_4_: ^1^H-NMR (250 MHz, C_6_D_6_): δ = 0.36 (s, 108 H, Si*Me_3_*), 2.15 (s, 54 H, tmeda), 2.3 ppm (s, 20 H, tmeda); ^13^C{^1^H}-NMR (63MHz, C_6_D_6_) δ = 2.61 (s; Si*Me_3_*), 5.21 (s, Si*Me_3_*), 46.04 (s, tmeda), 58.76 ppm (s, tmeda); ^29^Si-NMR (50 MHZ, C_6_D_6_) δ = 0.27–1.37 ppm (decet, ^2^*J*_Si-H_ = 6.5 Hz, Si*Me_3_*).

### 3.5. X-ray Structural Characterization

Crystals were mounted on the diffractometer at 150 K. The data were collected on a Bruker APEX II DUO diffractometer (Bruker AXS Inc., Madison, WI, USA) equipped with an IμS microfocus sealed tube and QUAZAR optics (Icoatec, Geesthacht, Germany) for monochromated MoKα radiation (λ = 0.71073 Å) and equipped with an Oxford Cryosystems cryostat (Oxford Cryosystems Ltd., Oxford, UK). A semiempirical absorption correction was applied using the program SADABS (Bruker AXS Inc., Madison, WI, USA). The structure was solved by Direct Methods and refined against F^2^ for all observed reflections. Programs used: SHELXS and SHELXL [37,38] within the Olex2 program package [39]. Both crystallized compounds **1** and **2** showed typical umbrella disorder at some of the Ge(SiMe_3_)_3_ substituents and were refined with a disorder model. Additionally, due to heavy disorder of solvent molecules in **1**, not all pentane molecules could be refined properly. Therefore, the SQUEEZE [40] routine was used to identify two voids of 224 Å³ per unit cell, which contain 41 electrons each, which leads to two more heavy disordered pentane molecules. The supplementary crystallographic data (for ccdc numbers, see below) can be obtained online free of charge at www.ccdc.cam.ac.uk/conts/retrieving.html or from Cambridge Crystallographic Data Centre, 12 Union Road, Cambridge CB21EZ; Fax: (+44)1223-336-033; or deposit@ccdc.cam.ac.uk.

#### 3.5.1. Sn_10_[Ge(SiMe_3_)_3_]_6_

C_54_H_162_Ge_6_Si_18_Sn_10_; M_r_ = 3048.10 g mol^−1^, crystal dimensions 0.277 × 0.253 × 0.128 mm^3^, space group *P*2_1_/*n*, *a* = 20.7885(5) Å, *b* = 26.5738(6) Å, *c* = 23.2212(6) Å, *β* = 93.2040(10)°, V = 12808.0(5) Å^3^, *Z* = 4, ρ_calc._ = 1.58 g cm^−3^, μ_Mo_ = 3.5 mm^−1^, 2θ_max_ = 52.808°, 26,7000 reflections measured, 26,232 independent reflections (R_int_ = 0.0379), absorptions correction: semiempirical (min./max. transmission 0.5818/0.7454), R_1_ (I ≥ 2σ) = 0.0281, wR2 (all) = 0.0706, Bruker APEXII diffractometer (MoKα radiation (λ = 0.71073 Å), 150 K). CCDC = 1832611.

#### 3.5.2. [Li(thf)_4_]{Sn_10_[Ge(SiMe_3_)_3_]_5_}

C_61_H_167_Ge_5_Si_15_Sn_10_LiO_4_; M_r_ = 2943.08 g mol^−1^, crystal dimensions 0.195 × 0.173 × 0.109 mm^3^, space group *P*2_1_/*n*, *a* = 17.2592(3) Å, *b* = 24.6275(5) Å, *c* = 30.3649(6) Å, *β* = 99.2340°, V = 12739.4(4) Å^3^, *Z* = 4, ρ_calc._ = 1.53 g cm^−3^, μ_Mo_ = 3.3 mm^−1^, 2θ_max_ = 61.082°, 345748 reflections measured, 38972 independent reflections (R_int_ = 0.0326), absorptions correction: semiempirical (min./max. transmission 0.6391/0.7461), R_1_ (I ≥ 2σ) = 0.0276, wR2 (all) = 0.0692, Bruker APEXII diffractometer (MoKα radiation (λ = 0.71073 Å), 150 K). CCDC = 1832610.

### 3.6. ^119m^Sn-Mössbauer Spectroscopy

The ^119m^Sn Mössbauer spectroscopic measurement of {Sn_10_[Ge(SiMe_3_)_3_]_4_}^2−^ and Sn_10_[Ge(SiMe_3_)_3_]_6_ was conducted with a Ca^119m^SnO_3_ source in the usual transmission geometry at 6 K. The sample was sealed in a thin-walled quartz container with a sample thickness of about 10 mg Sn cm^–2^. To reduce the K X-rays emitted by the ^119m^Sn Mössbauer source, a thin palladium foil (thickness 0.05 mm) was placed in front of the detector. The spectrum was measured over a period of 3 days. Fitting of the spectrum was done with the Normos-90 software package [41].

### 3.7. EDX

EDX analysis was performed by placing the cluster compounds on a graphite carrier into a HITACHI SU8030 scanning electron microscope (Hitachi High Tech Americas Inc. Schaumburg, IL, USA) with BRUKER-EDX (Bruker, Karlsruhe, Germany).

### 3.8. NMR

^1^H-, ^13^C{^1^H}-, and ^29^Si-INEPT-NMR measurements were done with a Bruker DRX-250 spectrometer (Bruker, Karlsruhe, Germany). All values are given in ppm against the external standard SiMe_4_.

### 3.9. Quantum Chemical Calculations

Quantum chemical calculations were carried out with the RI-DFT version [27] of the Turbomole program package (Turbomole GmbH, Karlsruhe, Germany) [24] and the TmoleX graphical user interface (Cosmologic GmbH, Leverkusen, Germany) [33] by employing the BP86 functional [25,26]. The basis sets were of SVP quality [28]. All model compounds are calculated as being structurally optimized. Partial charges of all atoms are calculated via an Ahlrichs–Heinzmann population analysis [29,30,31,32] (see Supplementary Materials for further details).

## 4. Conclusions

The disproportionation reaction of metastable Sn(I) halides proved to be a useful synthetic route to metalloid tin clusters, where the new Ge(SiMe_3_)_3_ ligand is used to obtain the metalloid cluster compounds Sn_10_[Ge(SiMe_3_)_3_]_6_ **1**, [Li(thf)_4_]{Sn_10_[Ge(SiMe_3_)_3_]_5_} **2**, and [Li(TMEDA)_2_]_2_{Sn_10_[Ge(SiMe_3_)_3_]_4_} **3**. The ten tin atoms within these clusters are arranged in the form of a distorted centaur polyhedron being isostructural to the corresponding Hyp derivatives Sn_10_(Hyp)_6_, [Sn_10_(Hyp)_5_]^–^, and [Sn_10_(Hyp)_4_]^2–^. However, **1**, **2**, and **3** show electronic differences to their Hyp derivatives, as revealed by ^119m^Sn Mössbauer spectroscopy, and also show slightly different stabilities. The now-available metalloid tin clusters with different substituents can be used in further investigations to reveal the influence of the substituent on the reactivity of a metalloid tin cluster, where especially the metalloid tin cluster {Sn_10_[Ge(SiMe_3_)_3_]_4_}^2–^ is of central importance due to its open ligand shell and moderate yield synthesis (22%). Here, deeper insight into the formation mechanism of the recently obtained intermetalloid cluster [Au_3_Sn_18_(Hyp)_8_]^−^ might also be possible as now reagents with different substituents are available, which is a part of ongoing research in our laboratory.

## Figures and Tables

**Table 1:** Comparison of selected bond distances [pm] of the metalloid clusters Sn_10_[Ge(SiMe_3_)_3_]_6_ **1** and Sn_10_[Si(SiMe_3_)_3_]_6_.

	Sn_10_[Ge(SiMe_3_)_3_]_6_ 1	Sn_10_[Si(SiMe_3_)_3_]_6_
Sn1-Sn2	292.2	289.6
Sn1-Sn8	288.5	288.1
Sn2-Sn4	295.5	293.5
Sn3-Sn6	291.3	290.9
Sn4–Sn9	300.8	302.1
Sn5-Sn7	293.0	292.5
Sn5-Sn10	301.4	303.2
Sn7-Sn9	310.8	312.9
Sn1-Ge1	266.2	Sn1–Si1: 264.0

**Table 2:** Fitting parameters of the ^119m^Sn Mössbauer spectroscopic measurement at 5 K. *δ* = isomer shift, Δ*E*_Q_ = electric quadrupole splitting, *Γ* = experimental line width.

Compound	*δ* (mm∙s^–1^)	Δ*E*_Q_ (mm∙s^–1^)	*Γ* (mm∙s^–1^)	Area (Fixed)
Sn_10_[Ge(SiMe_3_)_3_]_6_	2.45(2)	1.59(5)	1.05(4)	60
	2.51(2)	0.54(4)	0.79(6)	40

**Table 3:** Comparison of the bond lengths in pm of the metalloid clusters {Sn_10_[Ge(SiMe_3_)_3_]_5_}^–^ and {Sn_10_[Si(SiMe_3_)_3_]_5_}^–^.

	{Sn_10_[Ge(SiMe_3_)_3_]_5_}^−^	{Sn_10_[Si(SiMe_3_)_3_]_5_}^−^
Sn1-Sn2	289.5	288.7
Sn3-Sn4	291.8	293.3
Sn3-Sn6	294.3	294.4
Sn4-Sn5	292.1	292.7
Sn5–Sn8	302.9	302.8
Sn7-Sn10	298.1	297.3
Sn8-Sn9	303.2	302.7
Sn9-Sn10	301.9	302.4

**Table 4:** Fitting parameters for the ^119m^Sn Mössbauer spectrum of **3** at 6 K; *δ* = isomer shift, Δ*E*_Q_ = electric quadrupole splitting, *Γ* = experimental line width. Parameters marked with an asterisk (*) were kept fixed during the fitting procedure.

Signal	*δ* (mm∙s^–1^)	Δ*E*_Q_ (mm∙s^–1^)	*Γ* (mm∙s^–1^)	Ratio (%)
A	1.93(2)	0.36(7)	1.04(6)	30 *
B	2.00(7)	0.8(1)	0.9(1)	10 *
C	2.712(6)	0.31(1)	0.71(1)	30 *
D	2.85(4)	1.05(9)	1.16(2)	30 *

## Supplementary Materials

Supplementary materials are available online. 1: results of quantum chemical calculations; 2: NMR spectra, Figures S5–S10; 3: Results of EDX measurements. 4. Bond length comparison of {Sn_10_[Ge(SiMe_3_)_3_]_4_}^2−^ and [Sn_10_(Hyp)_4_]^2−^.
