# Supplementary material for: LiGe(SiMe3)3: A New Substituent for the Synthesis of Metalloid Tin Clusters from Metastable Sn(I) Halide Solutions
Source: Molecules. 2018 Apr 26;23(5):1022. doi: 10.3390/molecules23051022 (PMC6102580; doi:10.3390/molecules23051022)
Supplement: Supplementary file 1 [file molecules-23-01022-s001.pdf]

- Supporting Information-

$LiGe(SiMe_3)_3$: a new Substituent for the Synthesis of Metalloid Tin Clusters from Metastable Sn^I Halide Solution

Mareike Binder[a], Claudio Schrenk[a], Theresa Block[b], Rainer Pöttgen[b] and Andreas Schnepf[a], *

[a] Institut für Anorganische Chemie, Universität Tübingen, Auf der Morgenstelle 18, D-72076 Tübingen, Germany. E-Mail: andreas.schnepf@uni-tuebingen.de

[b] Institut für Anorganische und Analytische Chemie, Universität Münster, Corrensstrasse 30, D-48149 Münster, Germany. E-mail: pottgen@uni-muenster.de

1. Quantum chemical calculations

1.1 $Sn_{10}(Ge(SiMe_3)_3)_6$

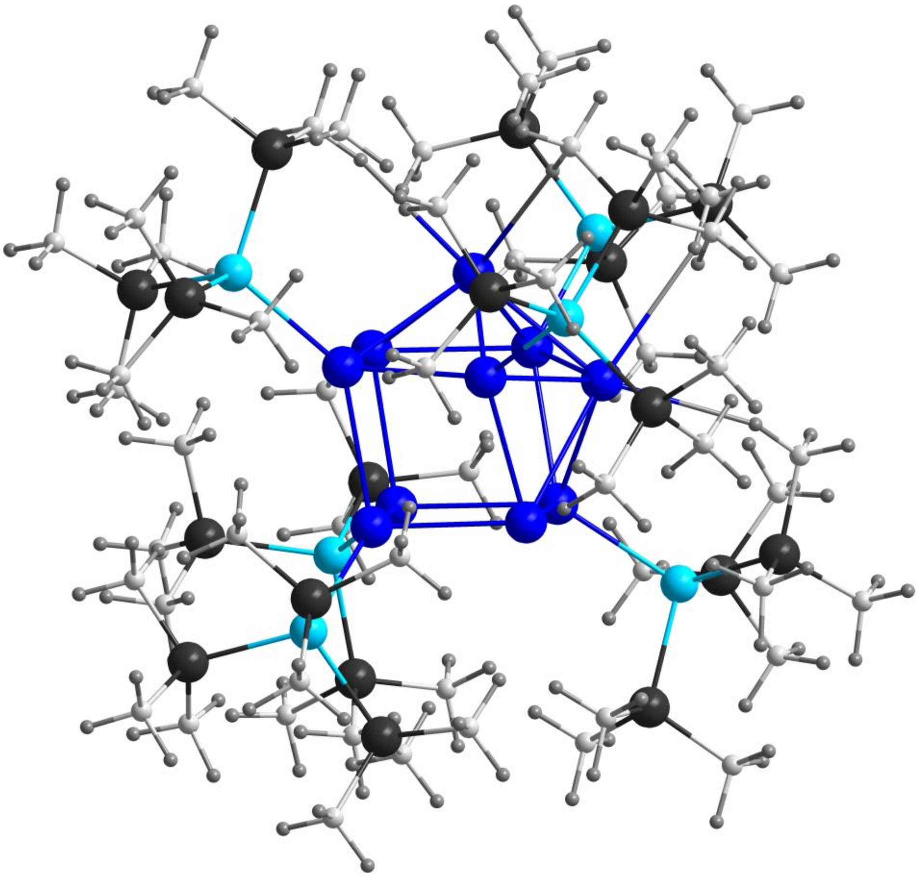

Figure S1: Optimized structure of $Sn_{10}(Ge(SiMe_3)_3)_6$

Point group used: C_1

Total energy: -19860.72629796513 H

HOMO-LUMO-gap: 1.065 eV

Atomic coordinates:

```
Sn 2.685579 -0.886251 -1.752562
Sn 0.583273 1.078538 -2.928665
Sn 0.590245 0.656415 2.518898
Sn -1.599796 -0.973296 1.049051
Sn -0.632291 2.850195 0.734189
Sn 3.200924 0.751323 0.774341
Sn -0.353689 -1.685885 -1.716764
Sn 1.916256 2.907851 -0.852512
Sn 1.401079 -1.928860 0.889424
Sn -2.018495 1.044357 -1.195072
Ge 1.480197 0.260799 5.139411
Ge -3.379659 -3.100215 1.366233
Ge 4.327753 -3.075675 -2.346731
Ge -0.291065 1.107265 -5.576807
Ge -2.499995 4.744871 1.541213
Si -0.345671 0.032881 6.735379
```

Si 5.360072 -2.805897 -4.537750
Si 2.790438 2.226049 5.751133
Si -2.306852 -5.139590 2.147268
Si -5.076158 -2.415946 2.976392
Si 3.185244 -5.227628 -2.292327
Si -1.262860 -1.024324 -6.250178
Si 2.938100 -1.676606 5.425877
Si 1.567064 1.666226 -7.049692
Si -1.969651 2.853242 -5.848936
Si -4.528383 -3.559167 -0.739486
Si -4.556818 3.635086 2.223849
Si -3.081005 6.102725 -0.394955
Si -1.765941 6.206656 3.339566
Si 6.119778 -3.102515 -0.689710
C 0.232735 0.479842 8.501472
C -1.794300 1.181607 6.289854
C 1.666486 3.755310 5.933024
C -4.293481 -1.652517 4.534627
C -3.636097 2.366243 -5.063842
C -0.975185 -1.767236 6.763674
C 6.010755 -1.035061 -4.793252
C 2.911933 -5.799096 -0.494699
C 3.693511 1.946672 7.412913
C -6.259156 -1.138291 2.196196
C 2.200514 -3.272372 4.693346
C 4.116150 -3.230680 -5.920136
C -1.357796 4.482059 -5.075172
C 1.867849 3.550447 -7.085938
C 4.122586 2.611113 4.447296
C -0.764872 -5.545045 1.108473
C 0.051238 6.717869 3.090648
C 0.069267 -2.387367 -6.247608
C 1.506753 -5.159875 -3.189173
C -1.784648 -4.974388 3.973076
C -2.689426 -1.554694 -5.106721
C 4.647803 -1.361061 4.646059
C 1.238251 1.101801 -8.845288
C 7.113807 -4.728689 -0.845485
C 4.256831 -6.548965 -3.166381
C -4.166576 2.202663 3.412965
C 7.310077 -1.637069 -0.957600
C -5.708740 4.861637 3.132709
C -6.108243 -3.924252 3.537134
C 6.854979 -3.984806 -4.716260
C 3.154357 0.807361 -6.447272
C -6.105160 -4.582904 -0.385900
C -5.515753 2.931667 0.731272
C -3.433857 -4.582452 -1.918757
C -5.065610 -1.954309 -1.611492
C -3.463109 5.002540 -1.901673
C -1.932471 5.331680 5.026459
C 3.193092 -1.987435 7.296685
C -3.520494 -6.608942 1.997777
C -1.986692 -0.887159 -8.014237
C -4.641172 7.148497 -0.038349
C -2.270946 3.166388 -7.711491
C -1.661915 7.286497 -0.859732
C -2.822164 7.798626 3.400338
C 5.443338 -3.011895 1.086297
Ge 3.839600 4.831767 -1.409321
Si 4.923922 5.363362 0.707203
Si 2.977674 6.906351 -2.337228
Si 5.572243 3.939807 -2.863955
C 6.082694 3.955718 1.268119
C 5.980042 6.949184 0.541131
C 3.614118 5.671035 2.055572
C 2.225899 7.999826 -0.965723
C 1.643999 6.557326 -3.650930
C 4.383524 7.901891 -3.166231
C 5.016132 3.941617 -4.687033
C 7.161562 4.995497 -2.741349
C 6.013441 2.157181 -2.358126
H 2.946317 4.790211 2.179612
H 4.097162 5.875249 3.038731
H 2.972391 6.542514 1.801095
H 6.488899 7.165928 1.509495
H 5.358320 7.834545 0.282149
H 6.765500 6.845327 -0.239194
H 3.003670 8.339022 -0.246159
H 1.755955 8.907690 -1.409906
H 1.442715 7.460697 -0.389599
H 4.828161 7.351114 -4.024533
H 5.202053 8.134439 -2.449761
H 3.986577 8.868905 -3.554628
H 6.507819 4.185612 2.272418
H 5.548149 2.981471 1.343281
H 6.933132 3.823304 0.562761
H 2.048614 5.938545 -4.482471
H 1.261664 7.508855 -4.087432
H 0.778478 6.010244 -3.217109
H 6.974135 6.060519 -3.000912
H 7.598806 4.964905 -1.718957
H 7.929261 4.602203 -3.448131
H 5.817745 3.519414 -5.336341
H 4.102563 3.326280 -4.834660
H 4.793799 4.970286 -5.048250
H 6.385645 2.114036 -1.310734
H 5.129433 1.484058 -2.426609
H 6.807761 1.747201 -3.023114
H -4.867729 7.793982 -0.919074
H -5.530652 6.505604 0.144160
H -4.512127 7.810573 0.845243
H -2.633696 2.254141 -8.233970
H -3.043866 3.960162 -7.838309
H -1.347902 3.509575 -8.229008
H -6.582213 -4.453355 2.681891
H -6.920669 -3.589485 4.223783
H -5.483765 -4.660615 4.090179
H 1.204105 -3.500995 5.131413
H 2.874686 -4.134162 4.907385
H 2.078730 -3.201594 3.588777
H 1.019575 -6.162382 -3.172712

H 0.813900 -4.435691 -2.703669
H 1.622675 -4.852924 -4.252167
H 4.825928 1.760490 4.313124
H 3.673701 2.829401 3.454193
H 4.715217 3.502961 4.756869
H -6.404548 2.360960 1.087454
H -4.888355 2.239706 0.124326
H -5.881822 3.737418 0.057257
H -1.071931 -4.132160 4.114599
H -1.279445 -5.906865 4.316170
H -2.658396 -4.797682 4.638765
H -4.462226 -6.434178 2.562564
H -3.044959 -7.532223 2.403861
H -3.790895 -6.808502 0.937150
H 7.963167 -4.717990 -0.122908
H 6.483830 -5.615068 -0.609734
H 7.535056 -4.871658 -1.864237
H 3.188517 -2.622656 -5.843397
H 4.569285 -3.041111 -6.920748
H 3.818224 -4.302280 -5.881525
H -3.183624 -5.576454 -1.485591
H -3.969455 -4.755659 -2.880862
H -2.478477 -4.060393 -2.152311
H 3.378034 1.075473 -5.391533
H 4.030385 1.103841 -7.068520
H 3.058319 -0.299347 -6.497412
H 4.562718 -1.072249 3.574967
H 5.190389 -0.546947 5.175962
H 5.274219 -2.281034 4.703717
H 5.268654 -6.633015 -2.712530
H 3.762896 -7.545378 -3.084326
H 4.384926 -6.325335 -4.248521
H -1.284217 -2.106555 5.751166
H -1.859413 -1.855991 7.436361
H -0.195499 -2.468872 7.134535
H 7.262346 -3.913007 -5.751966
H 7.674431 -3.713274 -4.014572
H 6.583198 -5.046694 -4.530853
H -0.300861 -6.497313 1.455034
H -1.010967 -5.654157 0.029287
H -0.000332 -4.740852 1.199228
H -1.118922 4.355968 -3.996903
H -2.139500 5.271597 -5.159868
H -0.439988 4.852502 -5.580819
H 0.303600 1.542022 -9.257176
H 1.157386 -0.005170 -8.919662
H 2.082455 1.422891 -9.499304
H -6.834315 -1.575578 1.350141
H -6.993279 -0.780575 2.955029
H -5.708265 -0.252552 1.809975
H -3.656787 2.567928 4.330641
H -3.496956 1.457183 2.927356
H -5.099401 1.678667 3.723202
H 0.885915 -2.163962 -6.969441
H -0.376054 -3.369096 -6.530731
H 0.524979 -2.500096 -5.238619
H -3.905589 7.575649 3.516155
H -2.695428 8.410052 2.479530
H -2.508782 8.426240 4.267224
H -6.599053 -4.850794 -1.349232
H -6.840672 -4.008916 0.219871
H -5.880524 -5.528248 0.155063
H -2.200869 0.941417 5.284173
H -2.622927 1.071511 7.026988
H -1.482786 2.249407 6.282487
H 2.270452 4.647067 6.220194
H 1.148817 3.992264 4.977438
H 0.887411 3.608056 6.713533
H -3.681658 -0.756826 4.291584
H -5.087763 -1.336108 5.249891
H -3.636517 -2.381531 5.057395
H 2.242638 -2.269920 7.801084
H 3.911276 -2.828916 7.437763
H 3.605432 -1.097609 7.820113
H 0.726567 5.834675 3.094248
H 0.381153 7.402722 3.905785
H 0.196301 7.245270 2.121695
H 4.456230 1.140949 7.331064
H 2.997506 1.676794 8.236309
H 4.224403 2.882285 7.706895
H -4.326975 4.329468 -1.710349
H -3.706538 5.630651 -2.789482
H -2.595461 4.360546 -2.167984
H 3.878853 -5.987403 0.022771
H 2.324954 -6.746287 -0.475261
H 2.351317 -5.041484 0.097496
H 1.015396 4.090935 -7.553555
H 2.781486 3.778340 -7.682270
H 2.016393 3.968042 -6.066279
H -6.656682 4.349125 3.419430
H -5.973323 5.734271 2.496146
H -5.241129 5.250463 4.064347
H -5.593410 -2.193833 -2.563573
H -5.757952 -1.358447 -0.976774
H -4.196184 -1.304543 -1.857750
H -3.102386 -2.532761 -5.446728
H -3.517257 -0.812402 -5.108294
H -2.347443 -1.676516 -4.054801
H 4.910705 -2.051257 1.263757
H 4.730041 -3.837542 1.302089
H 6.280346 -3.077859 1.819563
H 6.772616 -0.663470 -0.917211
H 8.092347 -1.628574 -0.163446
H 7.826826 -1.699949 -1.940897
H 0.521514 1.551324 8.579424
H 1.101015 -0.133585 8.827418
H -0.599498 0.302268 9.222230
H -1.401902 7.972851 -0.023559
H -0.743765 6.721462 -1.132397
H -1.948292 7.912314 -1.736559
H -4.098124 1.505190 -5.595979
H -3.524305 2.083536 -3.992556

H -4.348956 3.221282 -5.119646
H -1.356323 4.381204 5.051073
H -1.544768 5.985637 5.841586
H -2.993398 5.091182 5.259928
H -2.839268 -0.173220 -8.048333
H -2.370739 -1.883891 -8.334689
H -1.233631 -0.555760 -8.761101
H 6.763804 -0.767264 -4.020042
H 6.497094 -0.946164 -5.792381
H 5.193977 -0.283606 -4.746516

Partial charges:

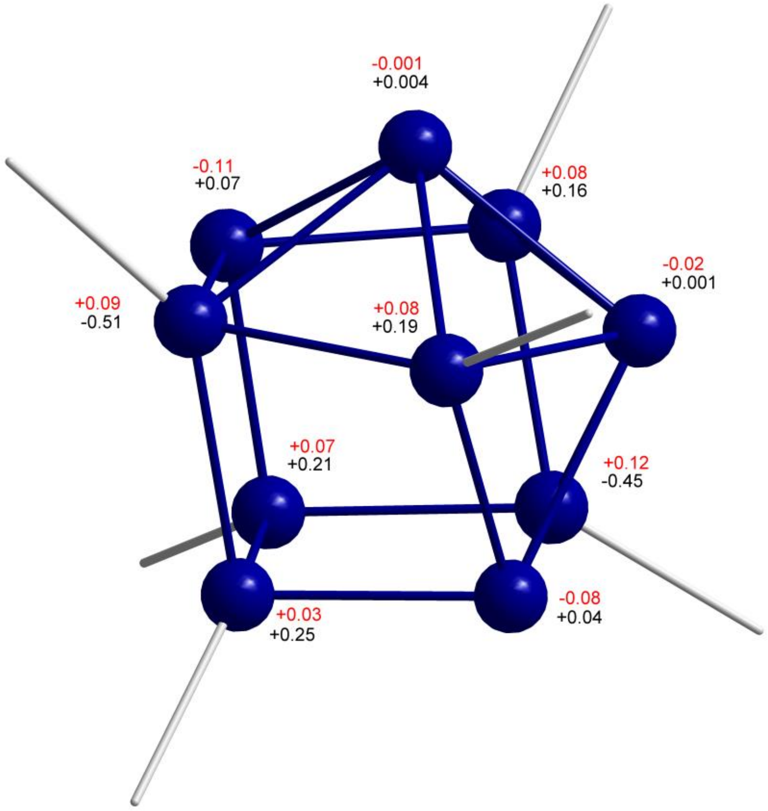

Figure S2: Comparion of the partial charges of the Sn atoms in $Sn_{10}(Si(SiMe_3)_3)_6$ (black) and $Sn_{10}(Ge(SiMe_3)_3)_6$ (red)

atom	charge
1 sn	0.0842
2 sn	0.0961
3 sn	0.1207
4 sn	0.0844
5 sn	0.0775
6 sn	-0.0809
7 sn	-0.0010
8 sn	0.0387
9 sn	-0.0225
10 sn	-0.1138
11 ge	-0.1948
12 ge	-0.1789
13 ge	-0.1839
14 ge	-0.1820
15 ge	-0.1932
16 si	0.4415
17 si	0.4422
18 si	0.4397
19 si	0.4432
20 si	0.4401
21 si	0.4439
22 si	0.4423
23 si	0.4426
24 si	0.4393
25 si	0.4449
26 si	0.4449
27 si	0.4425
28 si	0.4405

29	si	0.4413
30	si	0.4465
31	c	-0.3127
32	c	-0.3293
33	c	-0.3220
34	c	-0.3245
35	c	-0.3258
36	c	-0.3218
37	c	-0.3217
38	c	-0.3249
39	c	-0.3133
40	c	-0.3209
41	c	-0.3340
42	c	-0.3240
43	c	-0.3246
44	c	-0.3265
45	c	-0.3238
46	c	-0.3247
47	c	-0.3238
48	c	-0.3273
49	c	-0.3257
50	c	-0.3240
51	c	-0.3257
52	c	-0.3242
53	c	-0.3106
54	c	-0.3133
55	c	-0.3134
56	c	-0.3319
57	c	-0.3191
58	c	-0.3141
59	c	-0.3128
60	c	-0.3123
61	c	-0.3288
62	c	-0.3138
63	c	-0.3289
64	c	-0.3250
65	c	-0.3237
66	c	-0.3267
67	c	-0.3248
68	c	-0.3144
69	c	-0.3136
70	c	-0.3128
71	c	-0.3137
72	c	-0.3139
73	c	-0.3219
74	c	-0.3123
75	c	-0.3260
76	ge	-0.1944
77	si	0.4412
78	si	0.4420
79	si	0.4440
80	c	-0.3260
81	c	-0.3138
82	c	-0.3229
83	c	-0.3232
84	c	-0.3249

85	c	-0.3129
86	c	-0.3234
87	c	-0.3135
88	c	-0.3337
89	h	0.0773
90	h	0.0506
91	h	0.0657
92	h	0.0507
93	h	0.0631
94	h	0.0616
95	h	0.0623
96	h	0.0517
97	h	0.0748
98	h	0.0627
99	h	0.0612
100	h	0.0509
101	h	0.0522
102	h	0.0847
103	h	0.0601
104	h	0.0667
105	h	0.0497
106	h	0.0769
107	h	0.0619
108	h	0.0634
109	h	0.0509
110	h	0.0520
111	h	0.0737
112	h	0.0615
113	h	0.0675
114	h	0.0845
115	h	0.0547
116	h	0.0508
117	h	0.0624
118	h	0.0629
119	h	0.0615
120	h	0.0516
121	h	0.0628
122	h	0.0633
123	h	0.0516
124	h	0.0612
125	h	0.0651
126	h	0.0506
127	h	0.0924
128	h	0.0516
129	h	0.0847
130	h	0.0625
131	h	0.0684
132	h	0.0801
133	h	0.0511
134	h	0.0533
135	h	0.0868
136	h	0.0604
137	h	0.0787
138	h	0.0527
139	h	0.0608
140	h	0.0628

141 h	|	0.0520
142 h	|	0.0626
143 h	|	0.0517
144 h	|	0.0621
145 h	|	0.0626
146 h	|	0.0772
147 h	|	0.0517
148 h	|	0.0610
149 h	|	0.0588
150 h	|	0.0528
151 h	|	0.0840
152 h	|	0.0819
153 h	|	0.0525
154 h	|	0.0664
155 h	|	0.0843
156 h	|	0.0593
157 h	|	0.0519
158 h	|	0.0623
159 h	|	0.0516
160 h	|	0.0627
161 h	|	0.0739
162 h	|	0.0528
163 h	|	0.0624
164 h	|	0.0514
165 h	|	0.0619
166 h	|	0.0633
167 h	|	0.0523
168 h	|	0.0644
169 h	|	0.0833
170 h	|	0.0812
171 h	|	0.0517
172 h	|	0.0647
173 h	|	0.0620
174 h	|	0.0621
175 h	|	0.0516
176 h	|	0.0627
177 h	|	0.0506
178 h	|	0.0745
179 h	|	0.0674
180 h	|	0.0819
181 h	|	0.0530
182 h	|	0.0600
183 h	|	0.0532
184 h	|	0.0839
185 h	|	0.0620
186 h	|	0.0615
187 h	|	0.0511
188 h	|	0.0518
189 h	|	0.0627
190 h	|	0.0618
191 h	|	0.0841
192 h	|	0.0504
193 h	|	0.0661
194 h	|	0.0516
195 h	|	0.0754

196 h	|	0.0617
197 h	|	0.0797
198 h	|	0.0504
199 h	|	0.0651
200 h	|	0.0631
201 h	|	0.0513
202 h	|	0.0629
203 h	|	0.0801
204 h	|	0.0496
205 h	|	0.0644
206 h	|	0.0624
207 h	|	0.0637
208 h	|	0.0514
209 h	|	0.0682
210 h	|	0.0515
211 h	|	0.0802
212 h	|	0.0605
213 h	|	0.0530
214 h	|	0.0828
215 h	|	0.0627
216 h	|	0.0527
217 h	|	0.0796
218 h	|	0.0508
219 h	|	0.0617
220 h	|	0.0627
221 h	|	0.0519
222 h	|	0.0612
223 h	|	0.0878
224 h	|	0.0514
225 h	|	0.0638
226 h	|	0.0885
227 h	|	0.0827
228 h	|	0.0671
229 h	|	0.0520
230 h	|	0.0783
231 h	|	0.0508
232 h	|	0.0594
233 h	|	0.0622
234 h	|	0.0632
235 h	|	0.0513
236 h	|	0.0614
237 h	|	0.0760
238 h	|	0.0509
239 h	|	0.0581
240 h	|	0.0901
241 h	|	0.0516
242 h	|	0.0757
243 h	|	0.0519
244 h	|	0.0628
245 h	|	0.0611
246 h	|	0.0516
247 h	|	0.0634
248 h	|	0.0645
249 h	|	0.0494
250 h	|	0.0790

1.2. $Sn_{10}(Ge(SiMe_3)_3)_4^{2-}$

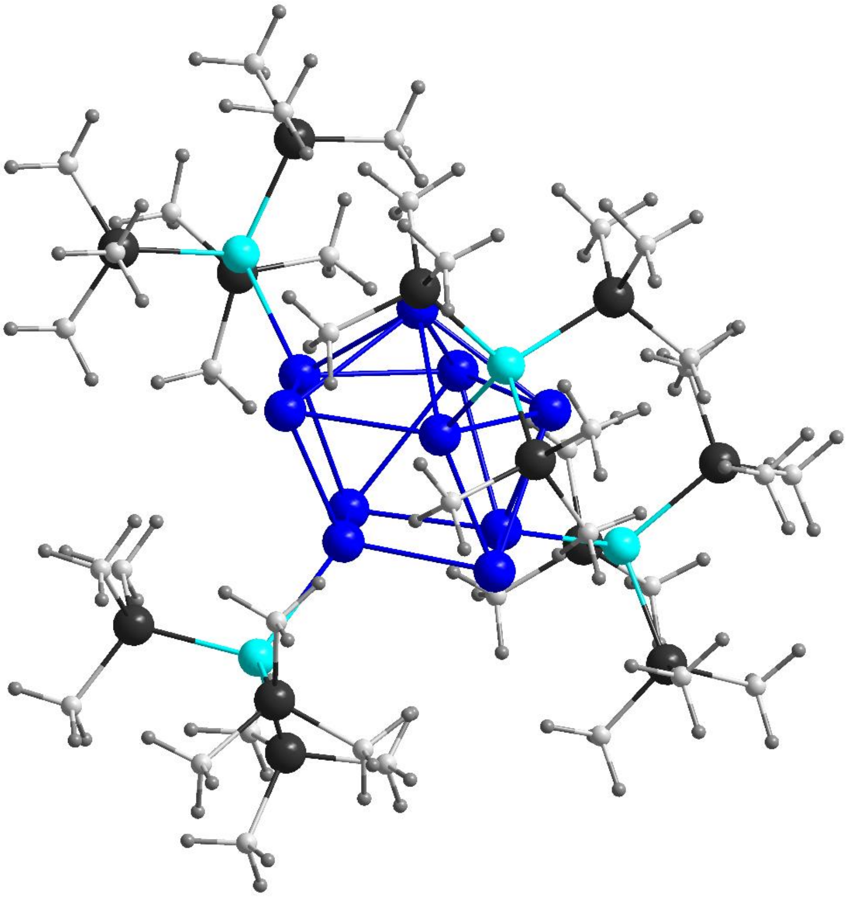

Figure S3: Optimized structure of $Sn_{10}(Ge(SiMe_3)_3)_4^{2-}$

Point group used: C_1

Total energy: -13252.12355283562 Hartree

HOMO-LUMO-gap: 1.657 eV

Atomic coordinates:

```
Sn -1.936760 0.479686 -1.786579
Sn -2.211025 0.537189 1.371057
Sn -1.550791 -2.151553 -0.121774
Sn 0.407119 -1.819248 -2.454619
Sn 1.073032 1.172857 -2.361326
Sn -0.472528 2.735926 -0.119141
Sn -0.018894 -1.724456 2.500748
Sn 0.662581 1.262710 2.415630
Sn 1.883026 -1.219988 0.157904
Ge -4.102690 -3.366468 -0.311194
Sn 2.703988 1.727516 0.178434
Ge 0.872786 2.954232 -4.555787
Ge 0.081512 3.098238 4.493949
Ge 4.113490 -2.830272 0.380750
Si -5.954693 -2.084479 -1.221157
Si -4.738943 -4.162607 1.892418
Si -3.795882 -5.314399 -1.729833
Si 1.859303 1.774272 -6.436208
Si -1.328120 3.718049 -5.243196
Si 2.252453 4.903198 -4.116547
```

Si 2.244805 3.846768 5.307410
Si -1.011120 1.926187 6.318272
Si -1.229754 5.074085 3.968293
Si 3.452065 -5.106277 0.883333
Si 5.551410 -2.024083 2.156336
Si 5.359438 -2.819606 -1.697165
C 2.884046 -5.270453 2.702746
C 4.864368 -6.383040 0.615500
C 1.989703 -5.628591 -0.227455
C 4.474556 -3.863536 -3.034264
C 5.513341 -1.037988 -2.365681
C 7.139163 -3.524450 -1.517276
C 3.735271 1.501569 -6.158907
C 1.677860 2.672732 -8.126753
C 1.061423 0.049622 -6.642113
C 2.705453 5.908344 -5.692520
C 1.361045 6.101655 -2.921574
C 3.900202 4.397063 -3.292093
C -0.220556 0.208662 6.593711
C -2.862469 1.646889 5.926363
C -0.924071 2.853746 8.000988
C -5.373757 -2.715040 2.969771
C -6.120223 -5.500755 1.871104
H -3.532588 -5.277041 3.817138
C -5.422118 -6.237377 -2.179030
C -2.651491 -6.597589 -0.885252
C -2.969846 -4.819156 -3.380450
C -1.269125 5.070374 -6.610049
C -2.298042 4.463307 -3.777521
C -2.360129 2.263288 -5.933389
C -6.143402 -0.403270 -0.336968
C -7.648134 -2.983396 -1.063224
C -5.678382 -1.726651 -3.079527
C 6.878484 -3.305595 2.697464
C 6.484070 -0.442161 1.619648
C 4.514414 -1.586549 3.698409
C 3.191845 2.405146 6.141293
C 3.327524 4.473287 3.862025
C 2.165491 5.271946 6.596095
C -2.873124 4.613625 3.113004
C -1.687583 6.114866 5.519920
C -0.267878 6.224183 2.780971
H 1.721647 6.190688 6.150641
H 3.189215 5.530660 6.958808
H 1.547047 4.993979 7.478731
H 2.680108 2.061627 7.068450
H 3.270254 1.531124 5.456548
H 4.226231 2.719740 6.416717
H 2.873568 5.360679 3.366867
H 4.344025 4.760752 4.221156
H 3.444917 3.688169 3.080363
H -1.473277 2.286561 8.790433
H -1.372025 3.870369 7.932906
H 0.129908 2.973222 8.339718
H -3.421536 2.608396 5.879247
H -3.340548 1.009194 6.706965
H -2.983675 1.137993 4.943028
H 0.856823 0.288916 6.860624
H -0.741124 -0.337514 7.415655
H -0.288727 -0.411690 5.670493
H -2.330796 5.532272 6.217513
H -0.783593 6.435180 6.084230
H -2.251935 7.031047 5.221868
H -3.514937 3.979529 3.764095
H -2.681845 4.040372 2.177712
H -3.447671 5.531995 2.845769
H 0.669660 6.607476 3.242970
H 0.008137 5.677168 1.850732
H -0.892944 7.101343 2.490402
C -3.241309 -4.931757 2.796936
H -2.827871 -5.803586 2.242404
H -2.419873 -4.186849 2.907289
H -5.557483 -3.050800 4.017627
H -6.324825 -2.294167 2.572632
H -4.626192 -1.889990 2.997746
H -7.040280 -5.132813 1.364264
H -5.784075 -6.416257 1.333715
H -6.394654 -5.798141 2.911686
H -4.714616 -1.189239 -3.232277
H -5.647280 -2.662096 -3.682199
H -6.497035 -1.084354 -3.481893
H -6.356424 -0.528470 0.747873
H -5.207419 0.193736 -0.422293
H -6.975046 0.187379 -0.789166
H -8.457316 -2.375464 -1.534813
H -7.920504 -3.140101 0.004969
H -7.634364 -3.980756 -1.556563
H -5.973805 -6.564846 -1.269532
H -5.202165 -7.142415 -2.794679
H -6.104056 -5.584099 -2.768780
H -2.002424 -4.296147 -3.201681
H -2.767449 -5.717688 -4.010161
H -3.612763 -4.125248 -3.966784
H -2.430575 -7.444301 -1.577344
H -1.682905 -6.134172 -0.591975
H -3.116799 -7.019050 0.034192
H -1.780724 5.349273 -3.346991
H -2.409449 3.715907 -2.959664
H -3.318205 4.778660 -4.101202
H -3.391681 2.600403 -6.191931
H -1.901338 1.826589 -6.848913
H -2.444739 1.453201 -5.173494
H -2.302986 5.355461 -6.920890
H -0.759961 5.989759 -6.242253
H -0.721558 4.721318 -7.513738
H 1.043894 5.569326 -1.996003
H 2.035128 6.939529 -2.624791
H 0.449992 6.542031 -3.385648
H 1.796642 6.251781 -6.235525
H 3.308219 6.808902 -5.423803
H 3.307964 5.295251 -6.400350
H 3.714329 3.859582 -2.333760

```
H 4.496190 3.719122 -3.942996
H 4.521300 5.296470 -3.068089
H 2.117340 3.694893 -8.098087
H 2.191829 2.097787 -8.934080
H 0.606884 2.775396 -8.413120
H 4.291577 2.465872 -6.148518
H 3.924730 0.993077 -5.187104
H 4.165319 0.866038 -6.968826
H 1.552521 -0.520545 -7.465899
H 1.151025 -0.547319 -5.705698
H -0.023802 0.127280 -6.876466
H 4.422734 -4.939025 -2.750903
H 5.009866 -3.790328 -4.010224
H 3.431585 -3.502987 -3.187568
H 4.505416 -0.583422 -2.500098
H 6.036434 -1.022132 -3.350695
H 6.076702 -0.384145 -1.663147
H 7.125162 -4.573246 -1.144289
H 7.669941 -3.514857 -2.499179
H 7.740153 -2.920543 -0.800371
H 5.766569 0.344901 1.292599
H 7.178751 -0.640114 0.772420
H 7.082517 -0.030549 2.466319
H 7.526713 -2.887600 3.504302
H 7.533721 -3.596270 1.845761
H 6.404567 -4.233942 3.089148
H 3.733700 -0.834422 3.442499
H 3.988936 -2.479154 4.105310
H 5.155208 -1.161792 4.506532
H 5.202113 -6.392310 -0.445480
H 4.520382 -7.413361 0.872478
H 5.749785 -6.148337 1.248138
H 2.505838 -6.299892 2.906746
H 2.060691 -4.553110 2.923562
H 3.714477 -5.062369 3.414604
H 1.634715 -6.653951 0.030770
H 2.270571 -5.619561 -1.304247
H 1.135791 -4.924027 -0.104760
```

Partial charges:

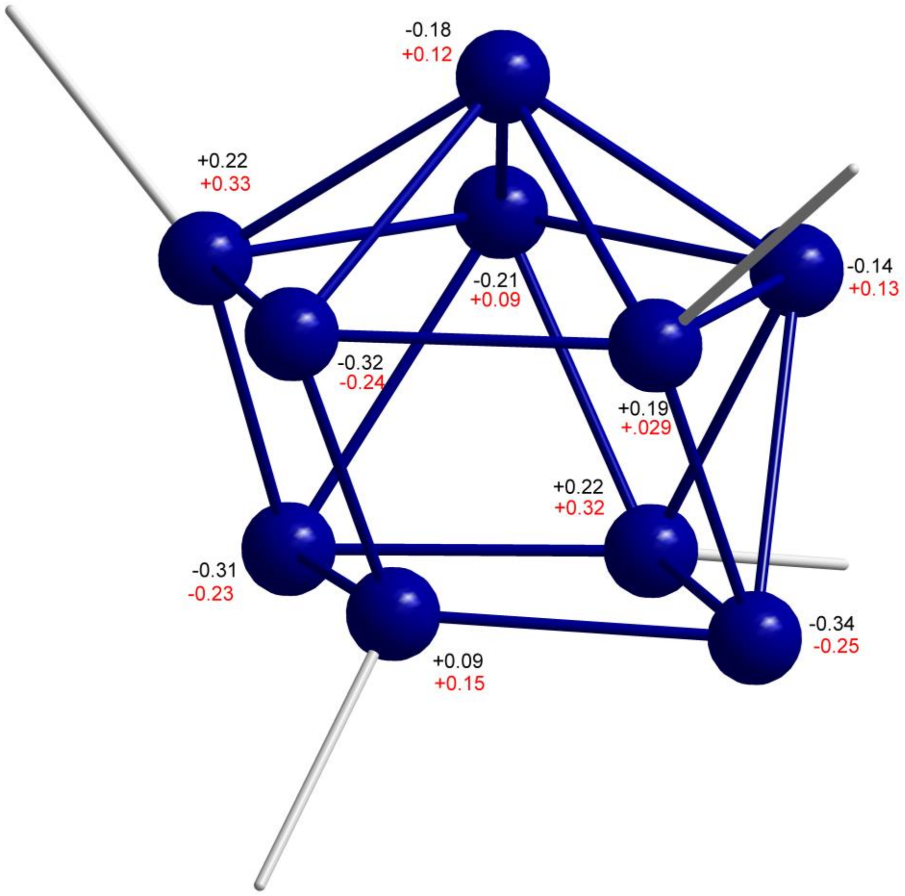

Figure S4: Comparion of the partial charges of the Sn atoms in $Sn_{10}(Si(SiMe_3)_3)_4^{2-}$ (black) and $Sn_{10}(Ge(SiMe_3)_3)_4^{2-}$ (red)

atom		charge
1 sn	|	-0.1316
2 sn	|	-0.1869
3 sn	|	0.1974
4 sn	|	-0.3370
5 sn	|	0.1784
6 sn	|	-0.1537
7 sn	|	-0.3175
8 sn	|	0.2027
9 sn	|	0.1180
10 ge	|	-0.2592
11 sn	|	-0.3268
12 ge	|	-0.2620
13 ge	|	-0.2569
14 ge	|	-0.1682
15 si	|	0.4335
16 si	|	0.4377
17 si	|	0.4314
18 si	|	0.4320
19 si	|	0.4339
20 si	|	0.4373
21 si	|	0.4315
22 si	|	0.4374
23 si	|	0.4338
24 si	|	0.4383
25 si	|	0.4386
26 si	|	0.4389
27 c	|	-0.3165
28 c	|	-0.3104
29 c	|	-0.3219
30 c	|	-0.3167
31 c	|	-0.3223
32 c	|	-0.3105
33 c	|	-0.3103
34 c	|	-0.3092
35 c	|	-0.3165
36 c	|	-0.3105
37 c	|	-0.3179
38 c	|	-0.3169
39 c	|	-0.3171
40 c	|	-0.3181
41 c	|	-0.3106
42 c	|	-0.3173
43 c	|	-0.3105
44 h	|	0.0311
45 c	|	-0.3092
46 c	|	-0.3105
47 c	|	-0.3160
48 c	|	-0.3100
49 c	|	-0.3173
50 c	|	-0.3185
51 c	|	-0.3173
52 c	|	-0.3100
53 c	|	-0.3183
54 c	|	-0.3103
55 c	|	-0.3167
56 c	|	-0.3219
57 c	|	-0.3105
58 c	|	-0.3165
59 c	|	-0.3091
60 c	|	-0.3178
61 c	|	-0.3099
62 c	|	-0.3177
63 h	|	0.0520
64 h	|	0.0311
65 h	|	0.0519
66 h	|	0.0495
67 h	|	0.0761
68 h	|	0.0319
69 h	|	0.0549
70 h	|	0.0300
71 h	|	0.0915
72 h	|	0.0318
73 h	|	0.0519
74 h	|	0.0512
75 h	|	0.0482
76 h	|	0.0330
77 h	|	0.0912
78 h	|	0.0523
79 h	|	0.0310
80 h	|	0.0954
81 h	|	0.0513
82 h	|	0.0521
83 h	|	0.0319
84 h	|	0.0547
85 h	|	0.0957
86 h	|	0.0320
87 h	|	0.0472
88 h	|	0.0904
89 h	|	0.0339
90 c	|	-0.3171
91 h	|	0.0520
92 h	|	0.0949
93 h	|	0.0330
94 h	|	0.0482
95 h	|	0.0902
96 h	|	0.0519
97 h	|	0.0511
98 h	|	0.0317
99 h	|	0.0905
100 h	|	0.0474
101 h	|	0.0336
102 h	|	0.0551
103 h	|	0.0942
104 h	|	0.0317

105 h	0.0319		138 h	0.0494
106 h	0.0513		139 h	0.0758
107 h	0.0521		140 h	0.0319
108 h	0.0518		141 h	0.0299
109 h	0.0312		142 h	0.0919
110 h	0.0520		143 h	0.0548
111 h	0.0920		144 h	0.0483
112 h	0.0299		145 h	0.0345
113 h	0.0545		146 h	0.0883
114 h	0.0321		147 h	0.0966
115 h	0.0760		148 h	0.0341
116 h	0.0493		149 h	0.0569
117 h	0.0549		150 h	0.0501
118 h	0.0936		151 h	0.0329
119 h	0.0318		152 h	0.0526
120 h	0.0336		153 h	0.0884
121 h	0.0474		154 h	0.0484
122 h	0.0906		155 h	0.0345
123 h	0.0319		156 h	0.0329
124 h	0.0513		157 h	0.0501
125 h	0.0520		158 h	0.0526
126 h	0.0910		159 h	0.0955
127 h	0.0330		160 h	0.0569
128 h	0.0480		161 h	0.0343
129 h	0.0519		162 h	0.0525
130 h	0.0317		163 h	0.0329
131 h	0.0510		164 h	0.0501
132 h	0.0951		165 h	0.0345
133 h	0.0519		166 h	0.0883
134 h	0.0310		167 h	0.0484
135 h	0.0518		168 h	0.0341
136 h	0.0312		169 h	0.0567
137 h	0.0521		170 h	0.0958

2. NMR

2.1 $Sn_{10}(Ge(SiMe_3)_3)_6$ 1

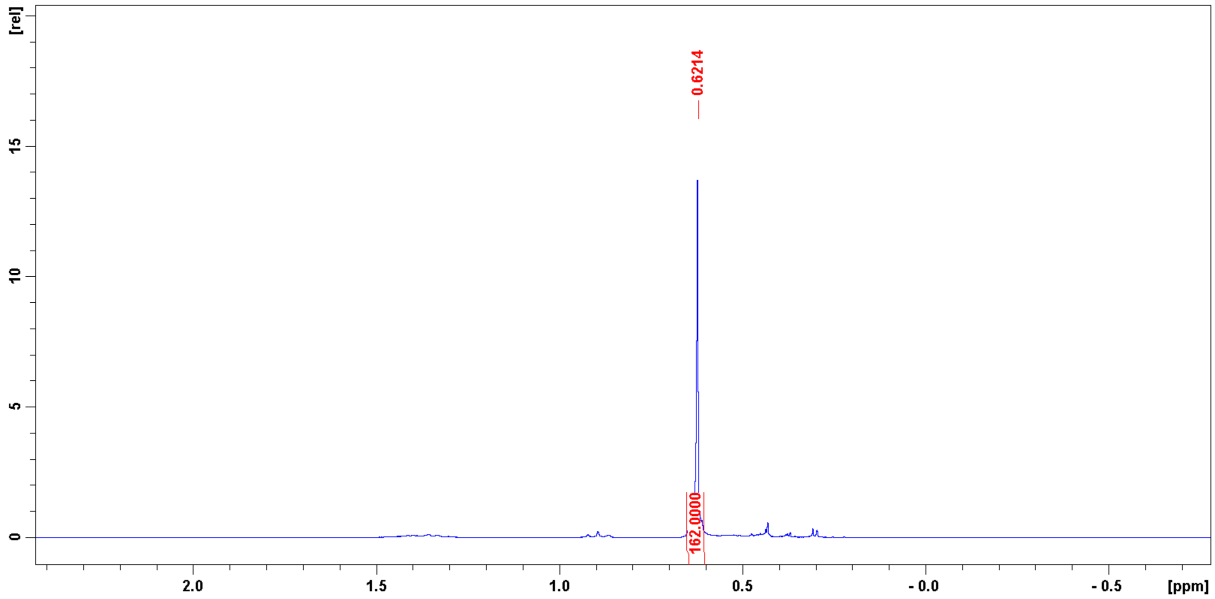

Figure S5: ^{1}H-NMR of **1** dissolved in C_6D_6. The signal at δ = 0.62 ppm shows the $Ge(Si\underline{Me}_3)_3$ ligand.

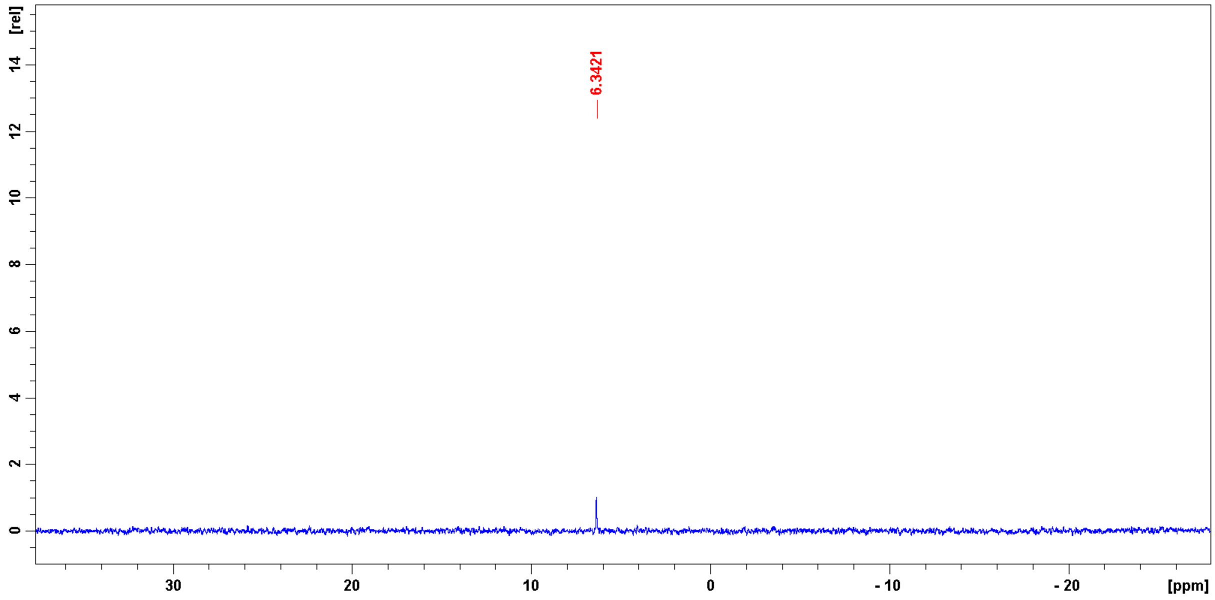

Figure S6: ^{13}C-NMR of **1** dissolved in C_6D_6. The spectra shows one signal at δ = 6.34 ppm for $Ge(Si\underline{Me}_3)_3$.

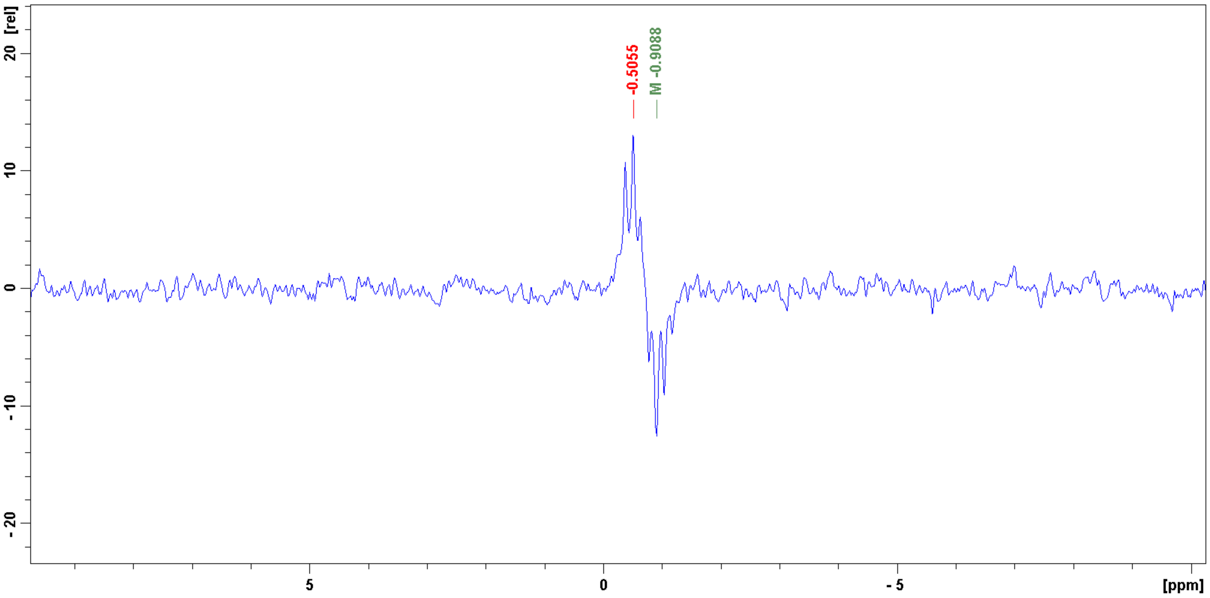

Figure S7: ^{29}Si-IneptND-NMR spectra of **1** dissolved in C_6D_6. The spectra shows a decet at δ = -0.13– -1.29 ppm for Ge(<u>*Si*</u>Me$_3$)$_3$ with $^2J_{Si\text{-}H}$ = 6.5 Hz.

2.2. $Sn_{10}(Ge(SiMe_3)_3)_4^{2-}$ 3

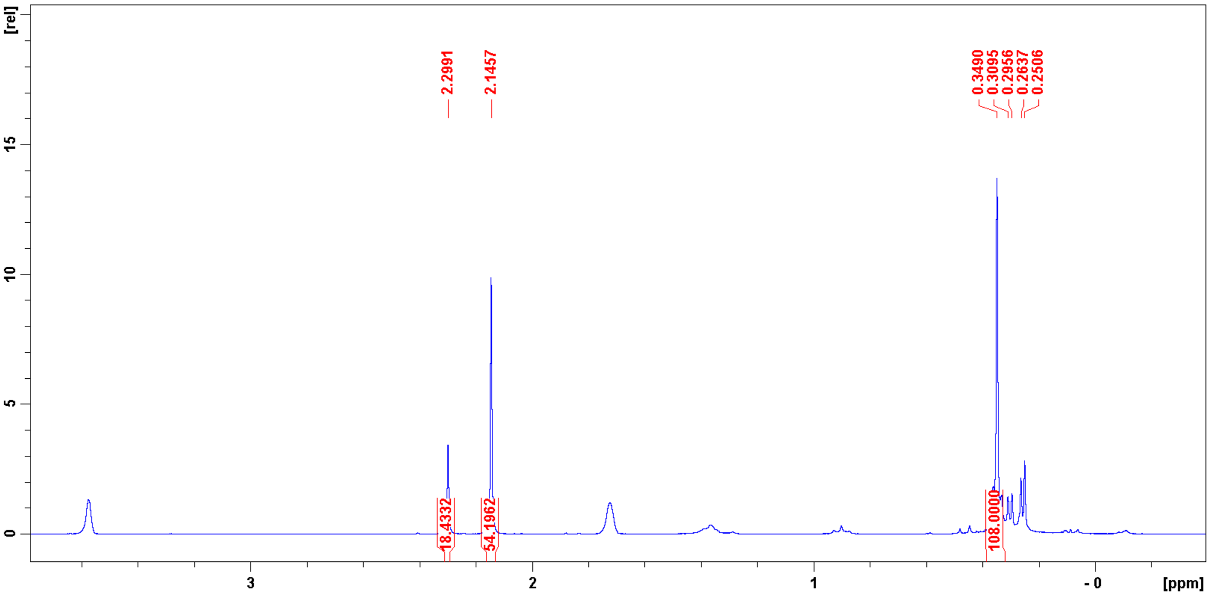

Figure S8: ^{1}H NMR spectra of the concentrated sample of **3**•2[Li(TMEDA)$_2$] in THF-d$_8$ directly after preparation. The signal at δ = 0.35 ppm represents the Ge(Si<u>*Me*</u>$_3$)$_3$, whereby the signals for tmeda can be found at 2.15 ppm and 2.30 ppm An impurity of Si(SiMe$_3$)$_4$ can be observed at 0.25/0.26 ppm.

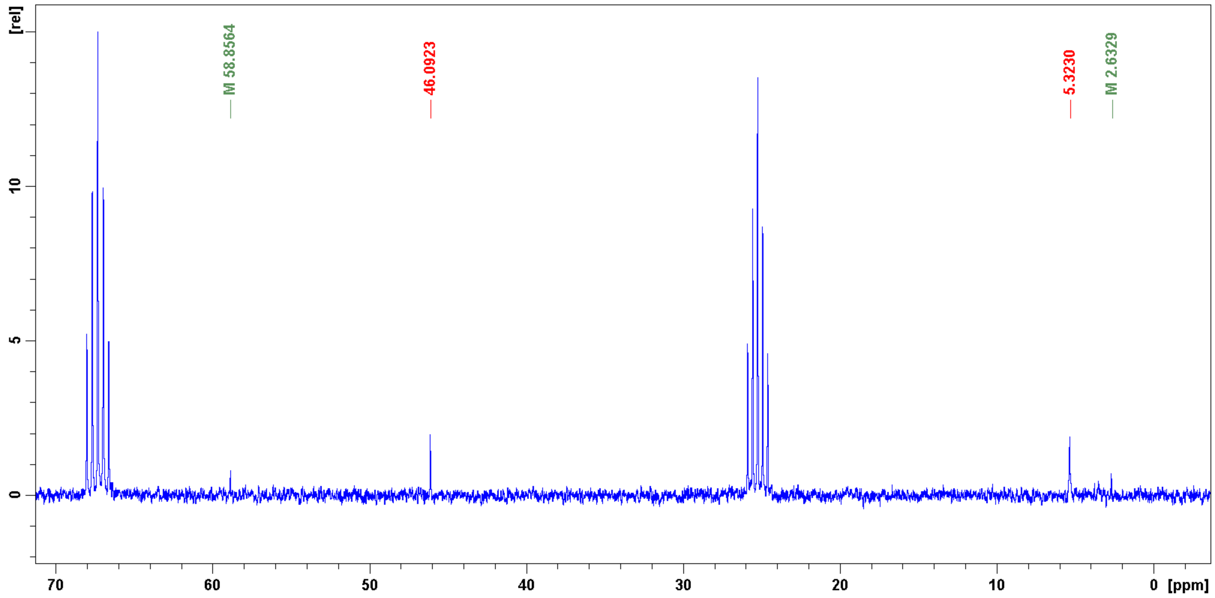

Figure S9: ^{13}C-NMR spectra of **3**•2[Li(TMEDA)$_2$] in THF-d$_8$. The signal at δ = 5.32 ppm represents the Ge(Si<u>*Me*</u>$_3$)$_3$ ligand. The signals for tmeda can be found at 2.15 ppm and 2.30 ppm. An impurity of Si(SiMe$_3$)$_4$ can be observed at 2.63 ppm.

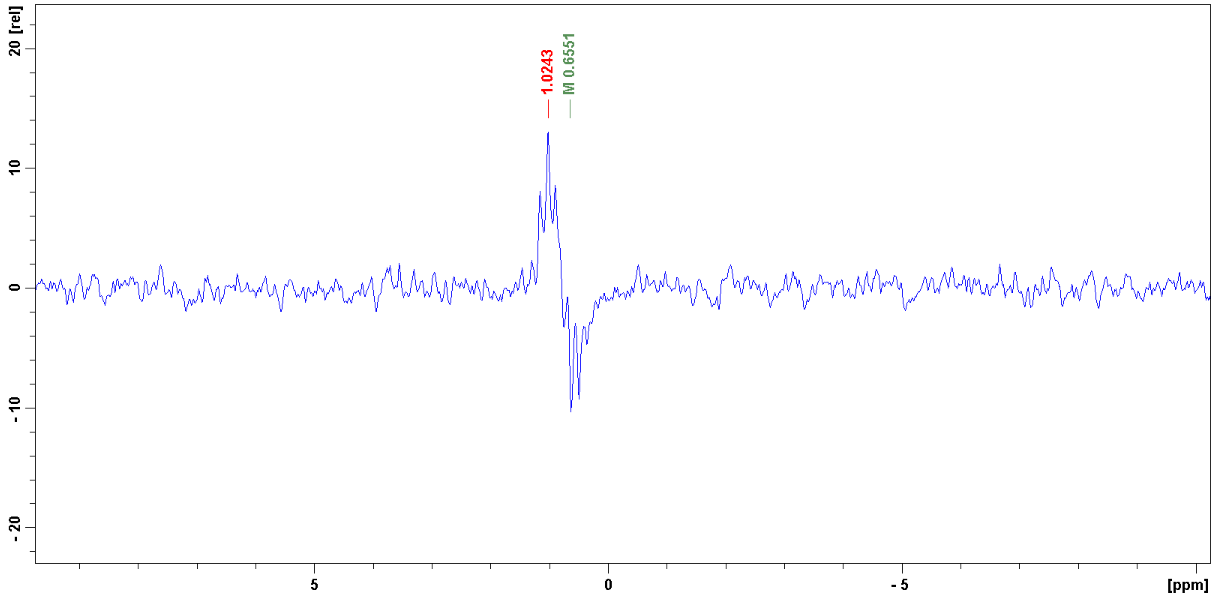

Figure S10: ^{29}Si-IneptND-NMR spectra of **3**•2[Li(TMEDA)$_2$] dissolved in THF-d$_8$. The spectra shows a decet at δ = 0.27 – 1.37 ppm for Ge(<u>*Si*</u>Me$_3$)$_3$ with $^2J_{\text{Si-H}}$ = 6.5 Hz.

3. Energy dispersive X-ray spectroscopy (EDX)

3.1. $Sn_{10}(Ge(SiMe_3)_3)_5^-$ 2

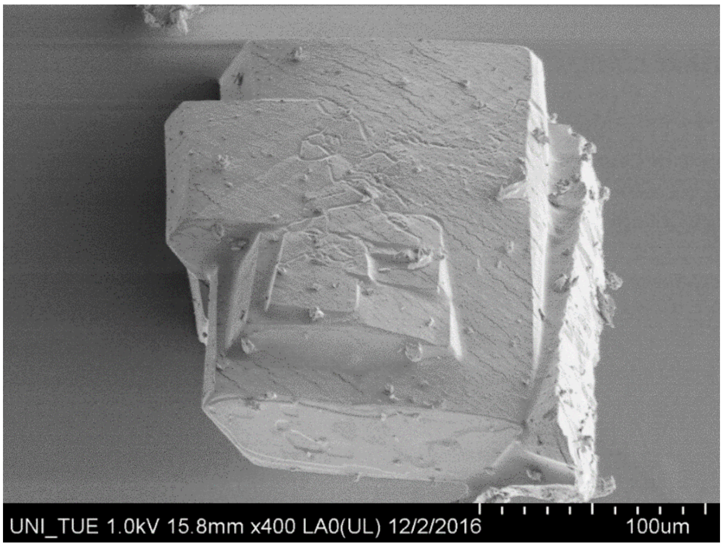

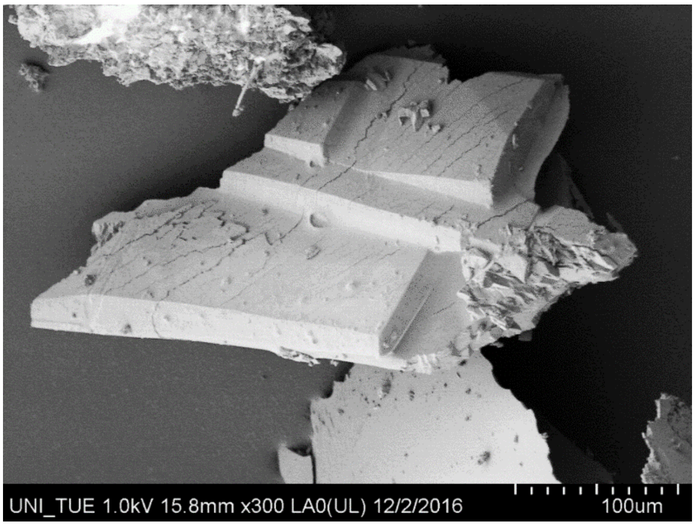

Figure S11**:** SEM-Image of a block shaped single crystal of **2** consisting of layers. The EDX measurements were performed at 7 different areas. Device: HITACHI SU8030 scanning electron microscope with Bruker-EDX

Table S1: Results of the EDX measurements of the crystallite at 7 different areas:

Element	Norm.Wt.%	Atoms calculated	Setpoint
Sn	56.89%	9,44	10
Ge	20.81%	5,65	5
Si	22.30%	15,65	15

4. Bond lenght comparison of $Sn_{10}[Ge(SiMe_3)_3]_4\}^{2-}$ 3 and $\{Sn_{10}[Si(SiMe_3)_3]_4\}^{2-}$.

Table S2: Comparison of the bond lengths in pm of the metalloid clusters $\{Sn_{10}[Ge(SiMe_3)_3]_4\}^{2-}$ **3** and $\{Sn_{10}[Si(SiMe_3)_3]_4\}^{2-}$.

$\{Sn_{10}[Ge(SiMe_3)_3]_4\}2-$ 3		$[Sn_{10}(Hyp)_4]2-$	
Sn1-Sn2	293.3	Sn1-Sn2	293.4
Sn3-Sn4	293.8	Sn3-Sn4	292.8
Sn6-Sn8	290.8	Sn6-Sn8	294.9
Sn3-Sn8	293.2	Sn3-Sn8	294.8
Sn5–Sn10	302.3	Sn5-Sn10	302.9
Sn4-Sn7	292.4	Sn4-Sn7	290.3
Sn9-Sn10	302.3	Sn9-Sn10	302.0
Sn8-Sn10	327.4	Sn8-Sn10	326.5
Sn6-Ge4	266.5	Sn6-Si4	263.1
